# Flavonoids influence key rhizocompetence traits for early root colonization and PCB degradation potential of *Paraburkholderia xenovorans* LB400

**DOI:** 10.3389/fpls.2024.1325048

**Published:** 2024-02-02

**Authors:** Elisa Ghitti, Eleonora Rolli, Lorenzo Vergani, Sara Borin

**Affiliations:** Department of Food, Environmental and Nutritional Sciences (DeFENS), University of Milan, Milan, Italy

**Keywords:** rhizosphere, beneficial bacteria, bioremediation, plant-microbe interactions, polychlorinated biphenyls, cry-for-help

## Abstract

**Introduction:**

Flavonoids are among the main plant root exudation components, and, in addition to their role in symbiosis, they can broadly affect the functionality of plant-associated microbes: in polluted environments, for instance, flavonoids can induce the expression of the enzymatic degradative machinery to clean-up soils from xenobiotics like polychlorinated biphenyls (PCBs). However, their involvement in root community recruitment and assembly involving non-symbiotic beneficial interactions remains understudied and may be crucial to sustain the holobiont fitness under PCB stress.

**Methods:**

By using a set of model pure flavonoid molecules and a natural blend of root exudates (REs) with altered flavonoid composition produced by *Arabidopsis* mutant lines affected in flavonoid biosynthesis and abundance (null mutant *tt4*, flavonoid aglycones hyperproducer *tt8*, and flavonoid conjugates hyperaccumulator *ttg*), we investigated flavonoid contribution in stimulating rhizocompetence traits and the catabolic potential of the model bacterial strain for PCB degradation *Paraburkholderia xenovorans* LB400.

**Results:**

Flavonoids influenced the traits involved in bacterial recruitment in the rhizoplane by improving chemotaxis and motility responses, by increasing biofilm formation and by promoting the growth and activation of the PCB-degradative pathway of strain LB400, being thus potentially exploited as carbon sources, stimulating factors and chemoattractant molecules. Indeed, early rhizoplane colonization was favored in plantlets of the *tt8 Arabidopsis* mutant and reduced in the *ttg* line. Bacterial growth was promoted by the REs of mutant lines *tt4* and *tt8* under control conditions and reduced upon PCB-18 stress, showing no significant differences compared with the WT and *ttg*, indicating that unidentified plant metabolites could be involved. PCB stress presumably altered the *Arabidopsis* root exudation profile, although a sudden “cry-for-help” response to recruit strain LB400 was excluded and flavonoids appeared not to be the main determinants. In the *in vitro* plant–microbe interaction assays, plant growth promotion and PCB resistance promoted by strain LB400 seemed to act through flavonoid-independent mechanisms without altering bacterial colonization efficiency and root adhesion pattern.

**Discussions:**

This study further contributes to elucidate the vast array of functions provided by flavonoids in orchestrating the early events of PCB-degrading strain LB400 recruitment in the rhizosphere and to support the holobiont fitness by stimulating the catabolic machinery involved in xenobiotics decomposition and removal.

## 1 Introduction

The plant microbiome acts as a reservoir of accessory functionalities that increase the fitness of the holobiont (Marasco et al., 2013; Rolli et al., 2015; Hassani et al., 2018; Trivedi et al., 2020). In particular, these microbial services are crucial when the soil is polluted by recalcitrant and poorly phyto-extractable xenobiotics that hamper plant growth and development (Franchi et al., 2016; Franchi et al., 2017; Correa-García et al., 2018). Plant competence to recruit a degradative microbiome and the enzymatic versatility of the associated microorganisms to catabolize persistent pollutants are essential features in rhizoremediation processes (Balloi et al., 2010; Vergani et al., 2017b; Simmer and Schnoor, 2022). Due to the advancement of metabolomics (Carper et al., 2022) and exo-metabolomics (Zhalnina et al., 2018), root chemistry is gaining increasing interest for the comprehension of the role of root exudation in tuning the recruitment, colonization pattern, structure, and functionality of the plant-associated microbiota (McLaughlin et al., 2023).

In addition to the exuded carbon-rich primary metabolites, plants secrete a vast array of secondary metabolites (Erb and Kliebenstein, 2020) that are responsible for the major shifts in the structure of soil microbial communities due to the presence of antimicrobial compounds, quorum sensing/quenching molecules, and co-metabolites that affect bacterial physiology (Pang et al., 2021).

Flavonoids constitute a broad class of specialized polyphenols that share the C_6_–C_3_–C_6_ basic structure and undergo a series of secondary modifications (e.g., glycosylation, methylation, acylation) that enable their classification into various classes like flavones, isoflavonoids, flavonols, and anthocyanins (Yonekura-Sakakibara et al., 2019). Flavonoids are among the most abundant compounds released by rhizodeposition (Chaparro et al., 2013; Wang et al., 2022): they represented 37% of total secondary metabolites released by *Arabidopsis thaliana* (Narasimhan et al., 2003) and reached a higher abundance compared with exuded primary metabolites in *Quercus ilex* upon water shortage stress (Singh et al., 2023). A metabolome investigation of the root exudate composition of *Panax notoginseng*, *Zea mays*, *Nicotiana tabacum*, and *Perilla frutescens* showed a high chemical diversity, with flavonoids being among the dominating compounds in terms of abundance and with differential patterns in terms of composition, contributing to the uniqueness of the exudation profile in the different plants (Shi et al., 2023).

Flavonoids are well characterized in legumes for their key role in rhizobial symbioses, facilitating the process of rhizobia recruitment and root architecture remodeling that leads to nodule formation, where the biological fixation of nitrogen takes place (Wang et al., 2022). Despite being acknowledged as inter-kingdom signaling molecules in the interactions between roots and plant-associated microbes (Ghitti et al., 2022), the role of flavonoids in non-symbiotic bacterial species is poorly investigated. Recently, a proteomics approach highlighted that apigenin and phloretin can be perceived by *Pseudomonas fluorescens* 2P24 through the TetR regulator system PhlH and that these flavonoids differently affected bacterial physiological traits involved in their establishment on the root system and in root colonization (Yu et al., 2020). Under nitrogen starvation, maize rhizodeposition was enriched of flavones, that positively selected for a higher abundance of *Oxalobacteraceae* in the rhizosphere microbiota. This shift in root bacterial communities indirectly supported plant growth and nitrogen uptake by remodeling root architecture with enhanced formation of lateral roots (Yu et al., 2021). Similarly, in the flavonoid hyperaccumulator *Arabidopsis* line *pap1-D* that overexpresses anthocyanins and flavonols, the *Aeromonadaceae* family showed a higher relative abundance in both rhizosphere and endosphere compared with Col-0 plants. Furthermore, flavonoids improved chemotaxis and motility of *Aeromonas* sp. H1 toward the root, enriching the rhizosphere with plant growth-promoting strains, finally able to boost plant dehydration resistance (He et al., 2022). Hence, these findings highlight a similarity in the role played by flavonoids as secondary metabolites involved in bacterial recruitment also in non-rhizobial species.

In bioremediation studies, flavonoids were described to act as inducers or co-metabolites to enhance the expression of the dioxygenases encoded by the *bph* operon for the aerobic degradation of polychlorinated biphenyls (PCBs) (Narasimhan et al., 2003; Toussaint et al., 2012). PCBs constitute a broad class of persistent organic compounds (POPs) that dramatically affect human health and ecosystems and whose removal from the environment, ideally through *in situ* sustainable practices, is of paramount importance and urgency (Di Guardo et al., 2017; Simhadri et al., 2020). Flavonoids are promising inducers of the microbial catabolism of PCBs, supposedly because of their similarity in structure with biphenyl (Pino et al., 2016). It is postulated that the PCB degradation pathway evolved primarily for the catabolism of plant secondary metabolites with a biphenyl-based backbone and that, due to a broad enzymatic specificity, this also allowed the degradation of structurally similar compounds like PCBs (Singer et al., 2004; Musilova et al., 2016; Uhlik et al., 2022). In agreement with this vision, co-metabolism drives the degradation of xenobiotic compounds in presence of plant secondary metabolites endowed with a structural affinity with the contaminants (Singer et al., 2003). Through these low substrate affinity enzymes, emerging pollutants of anthropogenic origin, only recently appearing in natural environments, can be degraded to low-molecular weight intermediates (Singer et al., 2004; Musilova et al., 2016; Uhlik et al., 2022). During co-metabolic growth in the presence of sodium acetate, isoflavone induced the expression of the *bphA* gene in *Rhodococcus erythropolis* U23A, resulting in an even higher degradation efficiency of 4-chlorobiphenyl than in the presence of biphenyl (Pham et al., 2015). Therefore, flavonoids may represent crucial exudates in the plant “cry-for-help” strategy in PCB-contaminated soil (Rolli et al., 2021). According to this ecological hypothesis, plants alter their root exudation pattern under stress aiming to recruit, feed, and sustain a wide variety of beneficial microorganisms, which provide useful functionalities to the plant to alleviate the detrimental injuries caused by biotic and abiotic stresses (Rolfe et al., 2019). Given PCB phytotoxicity and the poor detoxification systems of plants (Van Aken et al., 2010), the “cry-for-help” is hypothesized to be the strategy that plants employ to survive in polluted sites (Vergani et al., 2017a; Mapelli et al., 2022). Indeed, the depletion of flavonoid exudation in the *tt4 Arabidopsis* mutant affected the colonization and consequently the PCB degradation ability of *Pseudomonas putida* PLM2 (Narasimhan et al., 2003). Similarly, the PCB-degrading strain *Pseudomonas alcaliphila* JAB1 was able to metabolize flavone and flavanone through the activity of the biphenyl 2,3-dioxygenase and in parallel used a wide range of secondary metabolites, including flavonoids, as *bph* operon inducers (Zubrova et al., 2021). In wider terms, the identification and characterization of the chemical determinants able to induce PCB degradation would be particularly useful in the frame of tailor-made biostimulation strategies (Uhlik et al., 2013; Jha et al., 2015): providing the contaminated soil with inducer-rich amendments, also through waste biomass (Wang et al., 2023), or selecting plants with specific root exudation profiles would potentially enhance the recruitment of degrading microbial communities and increase the effectiveness of rhizoremediation.

This evidence supports the hypothesis that the role of flavonoids in affecting bacterial crosstalk with the plant host, in particular regarding the interactions with PCB-degrading bacteria, could be broader than supposed and still largely unknown. The aim of this work was, therefore, to go beyond the state of the art and elucidate the contribution of flavonoid molecules in affecting functional traits of rhizocompetence and degradation potential in the versatile PCB degrader strain *Paraburkholderia xenovorans* LB400 (Liang et al., 2014). Our experimental approach was developed along an increasing degree of complexity of flavonoid chemistry: (i) by administering pure compounds (naringin, naringenin, quercetin, flavone, and flavanone) to assess *in vitro* the involvement of flavonoids in stimulating bacterial rhizocompetence traits and in inducing the expression of PCB catabolic genes; (ii) by testing the effect of flavonoid imbalance in a natural complex mixture of root exudates released by the *Arabidopsis* mutant lines *tt4*, *tt8*, and *ttg* affected in flavonoid biosynthesis and exudation; and (iii) *in planta* in the interaction with the same mutant plants that differ in their root exudation pattern due to either the different genetic background or to the stress induced by growing in the presence of PCBs.

We observed a crucial role for flavonoid pure chemicals in boosting bacterial growth, attracting bacteria to the root system and stimulating the expression of PCB catabolic genes, indicating that flavonoids can play a prominent role during the early events of bacterial colonization. On the other hand, LB400 growth in the presence of the complex blend of root exudates from Arabidopsis mutant lines *tt4*, *tt8*, and *ttg*, together with the interaction of the bacterium with Arabidopsis plantlets of these backgrounds under control conditions and PCB-18 stress suggests flavonoid-independent mechanisms for the observed phenotypes, prompting that other unknown exudates are involved at later stages of bacterial colonization.

This work contributes to improve knowledge on the interactions between plants and *P. xenovorans* LB400, considered one of the most effective aerobic PCB degraders given its impressive ability to catabolize more than 20 PCB congeners, comprising some highly chlorinated ones (Chain et al., 2006). Burkholderiaceae have been described as important plant colonizers, represent a key component of the *Arabidopsis* microbiome, especially the floral one, and comprise beneficial bacteria able to support plant growth and resistance to abiotic stresses (Massoni et al., 2021; Pal et al., 2022). *P. xenovorans* LB400 has been used mainly in degradation studies with slurries or sediments (Payne et al., 2017; Bako et al., 2022): improving the knowledge on its association with plants could lead to more targeted and efficient phyto-rhizoremediation approaches for PCB clean-up in soil.

## 2 Materials and methods

### 2.1 Bacterial strain, plant material, culture media, and chemicals

*Paraburkholderia xenovorans* LB400 (DMSZ, Germany) was grown either in Tryptic Soy Broth (TSB, Merck, Darmstadt, Germany) or Luria-Bertani (LB) broth or in Mineral Medium Brunner (DSMZ, Germany) with a supplement of 30 mM sodium pyruvate as a carbon source. Bacterial cells were kept in glycerol stocks at −80°C and periodically revitalized on LB agar plates. When necessary, antibiotics were added at the following concentrations: 15 µg/mL gentamicin and 100 µg/mL rifampicin. The plant secondary metabolites (PSMs) used in the assays are flavonoids (Merck, Germany) and were solubilized in the respective solvents and prepared in 100× and 1,000× stocks. Flavone (2-phenyl-4H-1-benzopyran-4-one) and flavanone (2,3-dihydroflavone) were solubilized in acetone, naringin, and naringenin in methanol and quercetin in dimethyl sulfoxide (DMSO). The biphenyl crystals (Merck, Germany) were solubilized in acetone in a 0.5-M stock and PCB No. 18 (2,2′,5-trichlorobiphenyl, LGC Standards) was solubilized in acetone in a 200-mM stock. PCB-18 was selected to induce phytotoxic effect in *Arabidopsis* based on (i) literature data indicating that low-chlorinated PCBs are preferentially uptaken and assimilated by plant roots than highly chlorinated PCBs (Asai et al., 2002; Luo et al., 2020); (ii) a previous work demonstrating that this congener can indeed affect *Arabidopsis* growth (Bao et al., 2013); (iii) plant–microbe interaction study with PCBs are preferentially performed with single congeners rather than complex mixtures to make the phenotype analysis more straightforward (Subramanian et al., 2017; Wang et al., 2017); (iv) this molecule was retrieved in the historically PCB contaminated site Brescia-Caffaro in Italy, indicating its relevance also from an ecological point of view (Di Guardo et al., 2017). *Arabidopsis thaliana* ecotype L*er* and the mutants for flavonoid biosynthesis *tt4*, *tt8*, and *ttg* (NASC, Nottingham *Arabidopsis* Stock Centre) were the plants employed for root exudate collection and root colonization assays. Line *tt4* is a null mutant that does not produce flavonoids, whereas *tt8* and *ttg* overaccumulate in the root exudates flavonoid aglycones and flavonoid conjugates, respectively (Narasimhan et al., 2003). Seeds of *Arabidopsis* were surface sterilized under shaking conditions with a 0.05% SDS and 70% ethanol solution for 10 min and washed twice with 95% ethanol. The seeds were cultivated on half-strength Murashige and Skoog (1/2 MS) medium (2.2 g/L MS basal salt mixture, 0.5 g/L MES hydrate, pH 5.4) in square petri dishes.

### 2.2 Growth on flavonoids as unique carbon sources

Since *bph*-encoded enzymes originally evolved to catabolize plant secondary metabolites either as nutrients or for their detoxification (Singer et al., 2003; Singer et al., 2004), the ability of *P. xenovorans* LB400 to use pure plant flavonoids (flavone, flavanone, naringin, naringenin, and quercetin) as carbon sources was tested in liquid Mineral Medium Brunner (MMB) following the protocol reported by Zubrova et al. (Zubrova et al., 2021). Strain LB400 was cultivated overnight at 30°C on a shaker (150 rpm) in LB medium and cells harvested by centrifugation (10 min, 4,000 rpm). The cells were washed twice in physiological buffer (9 g/L NaCl) and resuspended at a final concentration of 0.025 OD_600_ in MMB containing 3-mM flavonoids in Erlenmeyer flasks. Solvents were used as negative controls by adding the corresponding volumes and 3 mM sodium pyruvate as positive control. Flavonoid solvents were evaporated in a sterile laminar hood for 30 min prior to addition of inoculated MMB. Strain LB400 was incubated for 6 days at 30°C on a rotatory shaker. To evaluate growth, bacterial cultures were plated in serial dilutions using the drop plate count method by spotting 10 µL on LB plates, which were incubated at 30°C for 3 days prior to colony counting. Each condition was tested with two independent replicates each with three technical replicates.

### 2.3 Bacterial growth assay in presence of flavonoids

To verify flavonoids’ ability to act as signaling molecules able to affect growth parameters of the bacterial strain, the protocol illustrated by Huang et al. (Huang et al., 2019) was adopted with some modifications. Briefly, strain LB400 was grown overnight on a shaker (150 rpm) at 30°C in 1/2 TSB liquid medium in ultrapure Milli-Q water up to 0.6 OD_600_, corresponding to the strain LB400 late-log growth phase. The cultures were centrifuged (5 min, 4,000 rpm), washed in physiological buffer, and diluted 1,000-fold in the various media used for the bioassay, consisting of the 1/10 TSB added with flavone, flavanone, naringin, or naringenin at final concentrations of 10 µM, 20 µM, 50 µM, and 100 µM. For the flavonoid quercetin, a maximum concentration of 70 µM was used to avoid the formation of an insoluble precipitate. The diluted cultures were then aliquoted (200 µL per well) into a transparent 96-well plate (VWR, USA). Cultures diluted in 1/10 TSB with only flavonoid solvents were used as negative controls. Abiotic controls were aliquoted as blanks in order to subtract the absorbance background given by the media. Each condition was tested in three biological replicates with, respectively, three technical replicates. Bacterial growth was monitored by measuring optical density at 600 nm every hour for 24/48 h using a 96-well plate reader (Tecan, Switzerland), keeping the plate incubated at 30°C and shaking for 7 s before each measurement. Bacterial relative growth increments were calculated as specified by Wang et al. (2019) by comparing the growth in the presence of flavonoids and the specific controls, as well as the log_2_ fold change at specific time points of the growth curves. Maximum growth rate was calculated as specified by Navarro-Perez et al. (Navarro-Pérez et al., 2022).

### 2.4 Analysis of bacterial swimming motility

Bacterial motility toward root exudates plays an important role in the colonization of the rhizosphere (Lugtenberg and Kamilova, 2009), and flavonoids were demonstrated to enhance flagellar motility in non-rhizobial strain (He et al., 2022). Following the indications of Kearns (Kearns, 2010) and Bartolini and Grau (Bartolini and Grau, 2019), 1/10 TSB medium with 0.25% (w/v) agar were prepared to evaluate swimming motility. The medium was autoclaved, briefly cooled, and supplemented with the flavonoids at concentrations 50 µM, 70 µM, and 100 µM before being poured into the plate. The appropriate solvent for each flavonoid molecule was added to the plate as negative control. The plates were dried with the lid for 2 h under laminar flow hood. Strain LB400 was grown overnight at 30°C on a shaker (150 rpm) in 1/2 TSB to an OD_600_ of 0.6. The culture was subsequently washed by centrifugation (5 min, 1,670*g*, 4°C) and diluted in physiological buffer to a concentration of 0.5 OD/mL. After drying the media, 2 µL of the bacterial suspension was spotted at the center of the plate and left to dry for 30 min with the lid, and then 10 min without the lid, under laminar flow hood. After 24 h of incubation at 30°C, the plates were scanned and the colony diameter measured in duplicate for each plate using ImageJ software (https://imagej.nih.gov). Each condition was tested with three biological replicates (two for quercetin) with at least four technical replicates.

### 2.5 Chemotaxis assay

Chemotaxis enhances bacterial capacity to acquire high-value nutrients (Colin et al., 2021) and is adopted by soil microorganisms to detect root exudates (Feng et al., 2021) that exert a chemoattractant role, as in the case of flavonoids (He et al., 2022). Chemotaxis was tested using the quantitative gradient plate assay proposed by Reyes-Darias et al. (Reyes-Darias et al., 2016). Minimal A gradient plate medium containing 0.25% (w/v) agar was poured in square Petri dishes after autoclaving. The plates were cooled with the lid for 3 h under laminar flow hood. 10 µL of 50 mM–100 mM pure flavonoids were spotted at the central line of the plate as chemoeffectors, whereas the solvents were used as negative controls and 100 mM sodium pyruvate as positive control for chemoattraction. Plates were further dried for 1 h under laminar flow hood and incubated overnight at 4°C to allow gradient formation. Strain LB400 was grown overnight at 30°C in 1/2 TSB and washed with physiological buffer as reported before for swimming motility assay. The bacterial culture was diluted to a concentration of 0.5 OD_600_, and, after drying the plates for 45 min, 2 µL of bacterial solution was spotted at 2.5 cm distance from the chemoeffector. The plates were dried for 20 min to allow the complete absorption of the bacterial drop and incubated at 30°C for 72 h. Results were collected by scanning the plates and measuring the colony radius toward (D1) and opposite to the chemoeffector (D2) using ImageJ software. The chemotaxis response index (RI) was then calculated as RI = D1/(D1+D2). Each condition was tested in at least three independent biological replicates, each with at least eight technical replicates.

### 2.6 *In vitro* biofilm formation assay

Biofilms are assemblages of bacterial cells embedded in an exopolysaccharide matrix that contribute to the bacterial adherence to the surface of the root system (Jin et al., 2019). The flavonoid apigenin was reported to stimulate biofilm formation by soil diazotrophic bacteria, promoting bacterial colonization of rice tissues and improving nitrogen fixation (Yan et al., 2022). Biofilm formation in the presence of flavonoids was therefore estimated *in vitro* by quantifying the bacterial cell adhesion to a solid surface (polystyrene 96-well plate, VWR, USA) using crystal violet (CV) staining, following the method applied by Yoshioka et al. (Yoshioka and Newell, 2016) with few adaptations. Briefly, strain LB400 was grown overnight at 30°C on a shaker (150 rpm) in liquid K10T-1 medium and 2 mL of bacterial culture was harvested and washed twice in physiological buffer. The bacterial solution was resuspended in fresh K10T-1, and 10 µL was added to each well, containing 200 µL of K10T-1 supplemented with 1-µM, 10-µM, and 100-µM flavonoids, to obtain a final concentration of 10^6^ cells/mL. Solvents were added to the medium as negative controls, and non-inoculated medium was aliquoted as blank. The plate was incubated statically for 2 days at 30°C. After incubation, OD_600_ of the bacterial cultures was measured using a 96-well plate reader (Tecan, Switzerland). The liquid culture was then carefully removed from the plate and the wells washed twice with phosphate-buffered saline (PBS). The cells adhering to the wells were stained for 15 min with 0.5% (weight/volume) crystal violet (CV) solution in 20% ethanol. CV solution was removed and the plate rinsed twice with distilled water and air dried for 15 min. The remaining CV was solubilized for 30 min using 200 µL/well of 97% ethanol, and OD_600_ was measured using a 96-well plate reader (Tecan, Switzerland). The CV optical densities obtained were then normalized using OD_600_ values measured previously for bacterial growth. Each condition was tested with three independent replicates.

### 2.7 Induction of the biphenyl degradative pathway in the LB400 strain by flavonoids

To assess if flavonoids act as potential inducers of the biphenyl catabolic pathway in strain LB400, an induction assay was performed. Adapting the protocol used by Pham et al. (Pham et al., 2015), LB400 was grown overnight in MMB supplemented with 30 mM sodium pyruvate on a rotatory shaker at 30°C. Aliquots of the bacterial culture (800 µL) were then used to inoculate glass vials containing 40 mL MMB supplemented with 30 mM sodium pyruvate only (as a control) or 30 mM sodium pyruvate plus 40 µL of flavonoids or biphenyl (positive control for *bphA* induction) to obtain a final concentration of 100 µM. The solvents in which flavonoids were dissolved were added in the same amount (40 µL) as negative controls in the absence of the putative inducer. All conditions were tested in triplicates. The vials were incubated at 30°C on a rotatory shaker, and for each condition, 1 mL of culture was sampled in triplicate when the bacterial cultures reached the mid-log phase (OD_600_ = 0.4-0.5), around 8 h after the start of the induction. Bacterial culture aliquots were then pelleted by centrifugation at 4,000 rpm at 4°C for 5 min. The pellet obtained was stored at −20°C for subsequent RNA extraction steps.

### 2.8 Total RNA extraction and RT-qPCR on the *bphA* gene

The induction of the oxidative biphenyl catabolic pathway for strain LB400 was analyzed by quantification of the expression of the *bphA* gene via reverse transcriptase quantitative PCR (RT-qPCR). Total RNA was extracted using the NucleoSpin RNA kit (Macherey-Nagel, Germany) following the manufacturer’s protocol, and RNA was eluted in 20 µL of RNAse-free water. To digest any residual gDNA, RNA cleanup and concentration protocol was performed using the NucleoSpin RNA XS kit (Macherey-Nagel, Germany) following the manufacturer’s instructions. Concentration and purity ratios of the extracted RNA were measured using a spectrophotometer (BioSpectrometer, Eppendorf, Germany). Reverse transcription (RT) was then carried out on 1 µg of RNA using RevertAid First Strand cDNA Synthesis kit (Thermo Fisher Scientific, USA) following the manufacturer’s indications. The thermal protocol requires the incubation of the template RNA with oligo(dT) primer for 5 min at 65°C, followed by incubation on ice for 5 min. After adding the reaction mixture, RT requires an incubation at 42°C for 60 min and 70°C for 5 min. Reactions in the absence of template RNA (NTC) and in the absence of reverse transcriptase (−RTC) were performed as controls. The cDNA obtained from reverse transcription of the total RNA extracted was diluted 1:10 in ultrapure Milli-Q water and subsequently used for qPCR. qPCR was performed using the CFX Connect Real-Time PCR Detection System (Bio-Rad, USA) using the SsoAdvanced Universal SYBR Green Supermix (Bio-Rad, USA). The reaction volume was 12 µL containing 1 µL template cDNA (5 ng/µL) and 0.25 µM primers for the amplification of LB400 *bphA* gene (F: 5′-AAAAAGGGCTGCTTGATCCA-3′; R: 5′-CGGTTTCAGGCACATGACTCT-3′) as the gene of interest, or the reference 16S rRNA gene (F: 5′-GAATTGACGGGGGCCCGCACAAG-3′; R: 5′-AGGGTTGCGCTCGTTG-3′). Thermal protocol was set up as follows: 95°C (10 min), and then 40 cycles at 95°C (10 s) and 60°C (40 s). A control without the template cDNA was run, and all reactions were performed in triplicates. The relative abundance of *bphA* gene expression was obtained by subtracting the threshold cycle (Ct) of the reference gene to the Ct of *bphA* and obtaining ΔCt. The ΔCt of the *bphA* gene in the presence of the inducer was then further compared with the ΔCt in control conditions in the absence of the inducer. The relative expression value of *bphA* was then calculated as 2^−ΔΔCt^. The baseline *bphA* expression in the absence of inducers is therefore represented by the relative expression value of 1 in the resulting graph.

### 2.9 *In vitro* growth assay on *Arabidopsis* root exudates collected under PCB-18 stress

The ability of strain LB400 to exploit plant root exudates, released under different conditions (differential abundance of flavonoids, control conditions, PCB-18 stress), was evaluated. For root exudate collection, around 30 surface-sterilized seeds of the *Arabidopsis thaliana* L*er* wild-type (WT) genotype, of the null mutant for flavonoid production *tt4* or of the flavonoid overproducers *tt8* and *ttg*, were cultivated on 1/2 MS liquid medium supplemented with 1% sucrose for 11 days. The medium was subsequently removed, the plants were washed twice with 1/2 MS, and then 10 mL fresh 1/2 MS liquid medium was added, supplemented with either 70 µM PCB-18 to induce the stress or acetone as untreated control. Root exudates from three biological replicates were collected and filtered at day 7 after the induction of the stress and stored at 4°C. Strain LB400 was inoculated in 1/2 TSB medium and incubated overnight at 30°C on a shaker (150 rpm). The next day, cells were collected and washed twice in physiological buffer by centrifugation (5 min, 4,000 rpm). Cells were inoculated in triplicates in a 96-well plate at a final concentration of 10^5^ cells/mL, with the previously collected root exudates used as culture media. 1/2 MS media containing only 70 μM PCB-18 or acetone were used as negative controls without plant root exudates. The plate was incubated on a rotatory shaker at 30°C for 3 days, and then the bacteria were re-isolated and quantified by plating serial dilutions using the drop plate count method, obtaining the number of CFUs/mL.

### 2.10 Generation of a fluorescence-labelled LB400 strain

To allow fluorescence microscopy observations of root colonization, LB400 strain was labeled with a constitutive mScarlet-I fluorescent protein via conjugation, adapting the protocol illustrated by Schlechter and Remus-Emsermann (Schlechter and Remus-Emsermann, 2019) for chromosomic insertion. The two strains used were *E. coli* S17-1 as donor strain, containing the Tn5 transposon delivery plasmid pMRE-Tn5-145 (Addgene, USA) expressing mScarlet-I, and a rifampicin-resistant strain LB400 (Rif^R^ LB400) as recipient strain. The strains were grown in LB medium as illustrated by the protocol: *E. coli* was used at a concentration of 0.5 OD_600_, whereas different growth phases were evaluated for the recipient strain, and 0.2 OD_600_ (early-log phase) was selected for its higher efficacy for strain LB400 conjugation. The bacterial cultures were then mixed at 1:5 (donor:recipient) ratios using a concentration of 10^9^ cells/mL of donor strain. Bacterial mating was carried out on nitrocellulose filter on an LB plate incubated a 30°C for 1 h. Trans-conjugants were gently resuspended from the filters using PBS and plated on LB containing 100 µg/mL rifampicin and 15 µg/mL gentamicin as selecting antibiotics. The trans-conjugants were re-streaked and single colonies tested for donor cell contamination via PCR using ITS primers (ITS-F: 5′-GTCGTAACAAGGTAGCCGTA-3′; ITS-R: 5′-GCAAGGCATCCACC-3′). Tn5 insertion in the recipient cells was then confirmed by multiplex PCR as described by Schlechter and Remus-Emsermann (Schlechter and Remus-Emsermann, 2019) using the primers FWD_Tn5_gt (5′-CTGAGTAGGACAAATCCGCCG-3′), REV_Tn5_gt (5′-GCCTCGGCAGAAACGTTGG-3′), FWD_Tn5/7_gt (5′-ATGGTGAGCAAGGGCGAG-3′) and REV_Tn5/7_gt (5′-CAACAGGAGTCCAAGCTCAG-3′). The phylogenetic identity of the trans-conjugant LB400 strains was then confirmed via Sanger sequencing of the 16S rRNA gene and the expression of mScarlet-I confirmed by fluorescence microscopy.

### 2.11 *In vitro Arabidopsis* root colonization assay in the presence of PCB-18

Strain LB400–*Arabidopsis* plantlet interaction was evaluated through *in vitro* assay under control conditions and under PCB-18 stress. Sterilized seeds were sown on 1/2 MS agar plates (9 g/L agar type E, Merck, Germany) containing *P. xenovorans* at a concentration of 2 × 10^5^ cells/mL, vernalized for 2 days at 4°C in the dark, and then placed vertically in a growth cabinet for 5 days (22°C, 50% humidity, long day conditions with light intensity of 120 µmol/m–150 µmol/m). Mock-inoculated plates (without the bacterial inoculum) were prepared by adding an equal volume of physiological buffer. Five DAG (days after germination), *Arabidopsis* plantlets were transferred onto fresh 1/2 MS plates containing 20 µM PCB-18 (treated) or an equal volume of acetone (untreated). The plates were incubated for a total of 14 days in vertical position in a growth cabinet. Root systems of the growing plantlets (the number varied depending on root dimension) were collected at DAT (days after transfer) 0, meaning the moment of the transfer to new plates, 7 and 14. The roots were placed in preweighed Eppendorf tubes and their fresh weight measured. The roots were then homogenized with a TissueLyser II (QIAGEN, Germany) using the following protocol: two cycles at 20 Hz frequency for 20 s and, after adding 900 µL of physiological buffer, two cycles at 15 Hz for 1 min. The smashed suspension obtained was used as 10^−1^ solution to prepare serial dilutions for the drop plate count method for cell counting on LB plates. After overnight incubation at 30°C, bacterial colonies were counted and the root colonization efficiency expressed as CFUs/mg root fresh weight. At DAT 7, the plates were scanned to measure root length (RL) and the number of secondary roots (NSR) using ImageJ software, and lateral root density (LRD) calculated as NSR/RL. At DAT 14 plant root, shoot and total fresh weights were measured. All measurements were performed on three independent experiments and on at least 12 plants per condition.

### 2.12 Fluorescence microscopy analysis

Root colonization analysis was performed by investigating the profile of colonization in different sections of the roots of plantlets colonized by strain LB400 labeled with mScarlet protein, developed as previously described. The analysis was performed on *Arabidopsis* roots 7 days after the transfer on acetone or PCB-18-supplemented plates. The analysis was performed at the microscopy platform Unitech NOLIMITS available at the University of Milan. The fluorescence emitted by the mScarlet-tagged bacteria colonizing *Arabidopsis* plantlets was observed at the stereomicroscope (Stereo Nikon SMZ) by scanning the root system with 15× magnification. For the red signal of mScarlet, the excitation and emission wavelengths used were, respectively, 561 nm and 570 nm–620 nm. For an optimized visualization of the mScarlet-labeled strain on the *Arabidopsis* root system, the maximum brightness of all epifluorescence microscopy images was adjusted to value 100 by using ImageJ software.

### 2.13 *In vitro* early root colonization assay

This assay was adopted to explore the ability of different patterns of flavonoid exudation to affect early events of root adhesion and colonization by strain LB400. Six-day-old *Arabidopsis* plantlets (L*er* WT, *tt4*, *tt8*, and *ttg*) were transferred individually to occupy a well in a 96-well plate with the root system positioned to ensure submersion in 300 µL of 1/2 MS liquid medium containing 10^7^ cells/mL of an overnight-grown bacterial culture of strain LB400. After 1 h of incubation at room temperature, the plantlets were briefly washed by dipping three times in physiological buffer, harvested, and pooled (n = 8) into a preweighed Eppendorf tube with a metallic bead. Root weight was measured, and the root systems were homogenized using a TissueLyser as previously specified. The bacterial colonization ability was estimated as CFUs/mg of root by plating serial dilutions on LB plates.

### 2.14 *In vitro* screening and quantification of strain LB400 plant growth promotion activities

#### 2.14.1 Indoleacetic acid production

Quantification of auxin produced by strain LB400 was performed as previously described (Bric et al., 1991). Briefly, strain LB400 was cultured on a shaker at 30°C for 72 h in LB medium supplemented with 500 µg/mL tryptophan. Optical density at 600 nm of the bacterial culture was measured, and 1 mL was harvested and centrifuged (10 min, 13,000 rpm) to collect the supernatant. 20 µL of orthophosphoric acid and 2 mL of Salkowski reagent (50 mL of 35% perchloric acid, 1 mL of 0.5 M FeCl_3_ solution) were added to the supernatant and incubated at room temperature for 20 minutes. The color intensity was measured using a spectrophotometer (OD_530_), and the quantity of auxin produced was obtained via interpolation with a standard curve obtained with indoleacetic acid (IAA) 10 µg/mL–100 µg/mL and normalized with the bacterial culture OD_600_.

#### 2.14.2 Production of volatile organic compounds that promote plant growth

Quantification of the plant growth promoting potential of volatile organic compounds (VOCs) released by strain LB400 was tested as in Ryu et al. (Ryu et al., 2003). Five-day-old *A. thaliana* seedlings were transferred on one side of a partition Petri dish containing solid 1/2 MS medium, whereas, on the other side, 20 µL of a bacterial suspension in physiological buffer at a concentration of 10^8^ cells/mL was spotted on LB medium. The bacterial suspension was obtained from strain LB400 grown overnight in LB medium and subsequently washed twice in physiological buffer by centrifugation (5 min, 4,000 rpm). 20 µL of physiological buffer without bacterium was used as negative control. Petri dishes were closed with Parafilm and incubated for 14 days in controlled conditions in a growth cabinet. Results of the plant growth promotion activity were registered by weighing the plant shoots (n = 6 from 5 independent plates and bacterial replicates).

#### 2.14.3 Production of siderophores

Bacterial production of siderophores was measured as reported by Cherif et al. (Cherif et al., 2015). An overnight culture of strain LB400 was washed in physiological buffer and resuspended at 0.001 OD_600_. 10 μL of the suspension was spotted on LB plates and incubated for 72 h at 30°C. Once grown, 12 mL of a CAS-blue agar solution was poured onto the LB plates, forming an overlay for the detection of the siderophore production, and incubated overnight at room temperature. Siderophore production was quantifiable as an orange halo around the bacterial colony, due to the change of color of the CAS-blue. Bacterial colony diameter (C) and siderophore halo diameter (S) were measured, and the siderophore production efficiency (SE) of the strain calculated as SE = (S-C)/C, as reported by He et al. (He et al., 2022).

#### 2.14.4 Formation of extracellular polymeric substances

Congo red (CR) assay for planktonic cells (Soo and Wood, 2013) was used to quantify the production of extracellular polymeric substances (EPS) as the amount of CR bound to the cells. Bound CR was calculated as the difference between the initial OD_490_, quantifying the CR present in the culture media and the remaining CR present in the supernatant after cell centrifugation after 3 and 6 h. The µg of CR bound to the cells was calculated via interpolation with a standard curve obtained with CR concentrations ranging from 0 µg/mL to 40 µg/mL and normalized with the bacterial culture density (OD_600_). The results were obtained from the average of five independent replicates.

#### 2.14.5 Measurement of ACC deaminase activity

ACC (1-aminocyclopropane-1-carboxylic acid) deaminase activity was measured as the quantity of α-ketobutyrate (α-KB) produced by the hydrolysis of ACC, which is a precursor of ethylene. The protocol illustrated in detail by Belimov et al. (Belimov et al., 2005) was followed and the amount of α-KB was quantified by measuring the OD_540_ and interpolating the value with a standard curve obtained with α-KB 10-100 µg/mL. α-KB concentrations obtained were normalized with the bacterial culture OD_600_. The results were obtained from the average of five independent replicates.

### 2.15 Statistical analyses

Statistical analyses were performed using R. Normal data were tested using one-way analysis of variance (ANOVA), followed by Tukey–Kramer *post-hoc* test for multiple comparisons (confidence interval 95%). For non-normal data, Kruskal–Wallis non-parametric test was adopted, followed by Dunn’s *post-hoc* test (confidence interval 95%). To compare distributions with small sample size (n < 30), Mann–Whitney non-parametric test was used (confidence interval 95%).

## 3 Results

### 3.1 Plant flavonoids promote *Paraburkholderia xenovorans* LB400 growth

**Figure 1:**
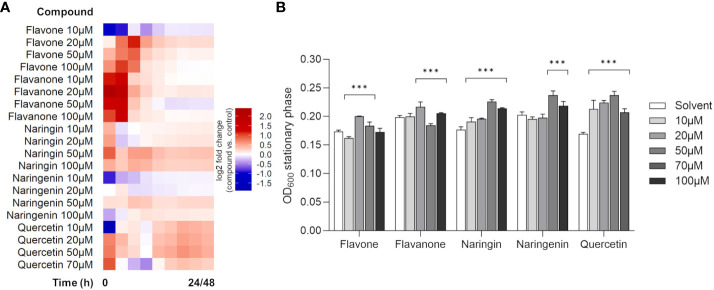
Effects of flavonoid compounds on the growth of *Paraburkholderia xenovorans* LB400. **(A)** Growth modulation activity of flavonoids supplemented as pure chemicals to 1/10 TSB liquid medium. The heatmap shows log_2_ fold change of strain LB400 treated with different concentrations (10 µM to 70 µM/100 µM) of the assayed flavonoids versus the respective solvent control at various time points over 24 h (48 h for quercetin only). The corresponding graphical growth curves are depicted in **Supplementary Figure 1**. **(B)** Bacterial biomass reached the stationary phase at 24 h (30 h for quercetin only) is expressed as OD_600_. The bars represent the average ± standard deviation of three independent replicates. Statistical analysis was performed using the Mann–Whitney test by comparing the flavonoid concentrations with the respective solvent control (***p ≤ 0.001).

To test whether the selected model flavonoids affected *P. xenovorans* LB400 growth, the bacterium was grown in liquid culture (1/10 TSB) amended with increasing concentrations (10 µM to 70 µM/100 µM) of flavonoids as pure compounds. Flavonoids are secondary metabolites, and therefore, this assay had the objective to verify their role as potential signaling molecules that could stimulate or hamper bacterial proliferation in a diluted carbon-rich medium. As indicated in the heatmap in **Figure 1A**, the assayed flavonoids selectively modulated the growth of strain LB400 in a concentration-dependent manner. In the presence of naringin and quercetin, bacterial growth was promoted at all assayed concentrations. Naringenin improved bacterial growth only at the highest concentrations of 50 and 100 µM, whereas for flavanone the growth promotion effect was observed at 20 µM and 100 µM and an inhibition effect was recorded at 50 µM. Flavone promoted strain LB400 proliferation at 20 µM and 50 µM (**Figure 1A** and **Supplementary Figure 1**). The flavonoid-mediated improvement in growth parameters is also mirrored in an increased bacterial biomass reached at the stationary phase, after 24 h/30 h of growth (**Figure 1B**). The higher relative growth increments compared with the control were recorded for 20 µM flavanone and flavone (+9.2% and +15.3%, respectively), for 50 µM naringin and naringenin (+27.8% and +17.3%, respectively) and for 50 µM quercetin (+40.1%) (**Figure 1B** and **Supplementary Figure 2**). The growth curves were elaborated to estimate the effect of flavonoid supplements on bacterial maximum growth rate (**Supplementary Table 1**). Under all assayed concentrations of naringin, strain LB400 exhibited a higher maximum growth rate compared with the control. An increase in this growth feature was observed for 20 µM flavone and 50 µM naringin, as well. Interestingly, quercetin supplements decreased bacterial maximum growth rate, but the achievement of a higher bacterial biomass could be attributed to a longer exponential growth phase and a delayed entrance in the stationary phase (**Figure 1**; **Supplementary Table 1** and **Supplementary Figure 1**). Given these findings, it was inquired if flavonoids could be exploited as carbon sources since *bph*-encoded dioxygenases originally evolved to catabolize plant secondary metabolites (Singer et al., 2003). By cultivating the strain in mineral medium supplemented by flavonoids as a unique carbon source, we observed that only naringin and naringenin could support bacterial growth (**Supplementary Figure 3**).

### 3.2 Flavonoids affect *Paraburkholderia xenovorans* LB400 chemoattraction, motility, and biofilm formation ability

**Figure 2:**
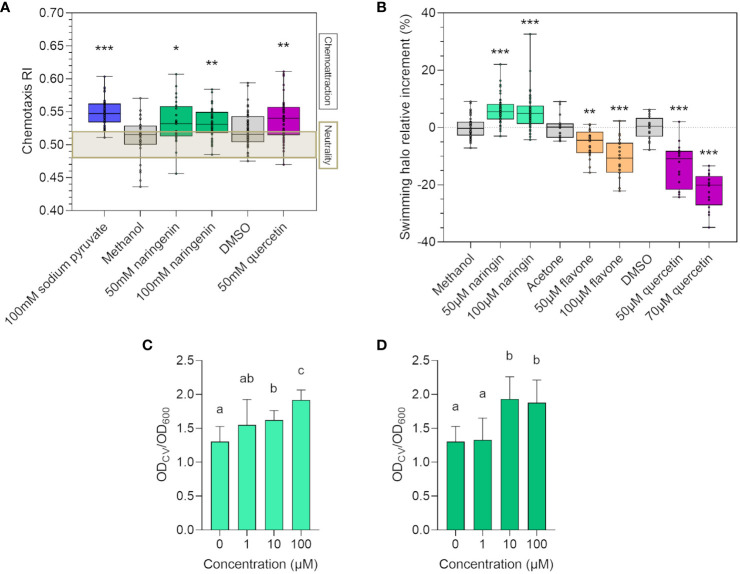
Flavonoids affect *P. xenovorans* LB400 chemotaxis, motility, and biofilm formation abilities. **(A)** Chemotaxis of strain LB400 toward flavonoids on gradient plates after 72 h of growth at 30°C is quantified by calculating the chemotaxis response index (RI). RI higher than 0.52 indicates chemoattraction, RI lower than 0.48 indicates repulsion, whereas 0.48 < RI < 0.52 indicates neutral behavior. Statistical analysis was carried out on at least three independent experiments using ANOVA followed by Tukey–Kramer *post-hoc* test (*p ≤ 0.05, **p ≤ 0.01, ***p ≤ 0.001). **(B)** Percentage of relative increase of the swimming halo diameter of strain LB400 was calculated as described by Wang et al. (Wang et al., 2019). Statistical analysis was carried out on at least three independent experiments using Dunn’s *post-hoc* test (*p ≤ 0.01; ***p ≤ 0.001). The graphs **(C, D)** report the relative biofilm formation ability of strain LB400 in the presence of the flavonoids naringin **(C)** and naringenin **(D)** expressed as the ratio between the OD_600_ value for crystal violet staining and the OD_600_ indicating bacterial growth, as described in the material and methods section. Error bars represent the standard deviation of three independent experiments. Statistical analysis was performed using Dunn’s *post-hoc* test, and letters indicate statistically different groups (p ≤ 0.05).

Chemotaxis and cell motility are considered essential features in the early phases of microbial colonization, leading to primary root surface attachment (Allard-Massicotte et al., 2016; Manner and Fallarero, 2018). Hence, we verified if flavonoids could modulate strain LB400 chemotactic responses, potentially influencing its root colonization ability. *In vitro* chemotaxis assay demonstrated that strain LB400 was attracted by 50 mM and 100 mM naringenin and by 50 mM quercetin (**Figure 2A**), whereas no statistically relevant effects were observed for the other flavonoid molecules assayed (data not shown). To evaluate if flavonoids regulate flagellar movement, an *in vitro* swimming assay was performed. While treatment with 50 µM and 100 µM naringin significantly increased the bacterial swimming halo, both 50 µM and 100 µM flavone and quercetin decreased strain LB400 swimming ability (**Figure 2B**). Biofilm formation is a fundamental feature for rhizospheric bacteria to ensure a stable attachment to the root surface (Zboralski and Filion, 2020): 10 µM and 100 µM naringin and naringenin promoted the ability of the bacterium to adhere and form a biofilm on the substrate (**Figures 2C, D**). These results suggest that the assayed flavonoids elicited different chemotactic motility and biofilm formation responses in *P. xenovorans* LB400, potentially influencing its recruitment by the plant through root exudation.

### 3.3 Flavonoids induce the expression of the *bphA* gene in strain LB400

**Figure 3:**
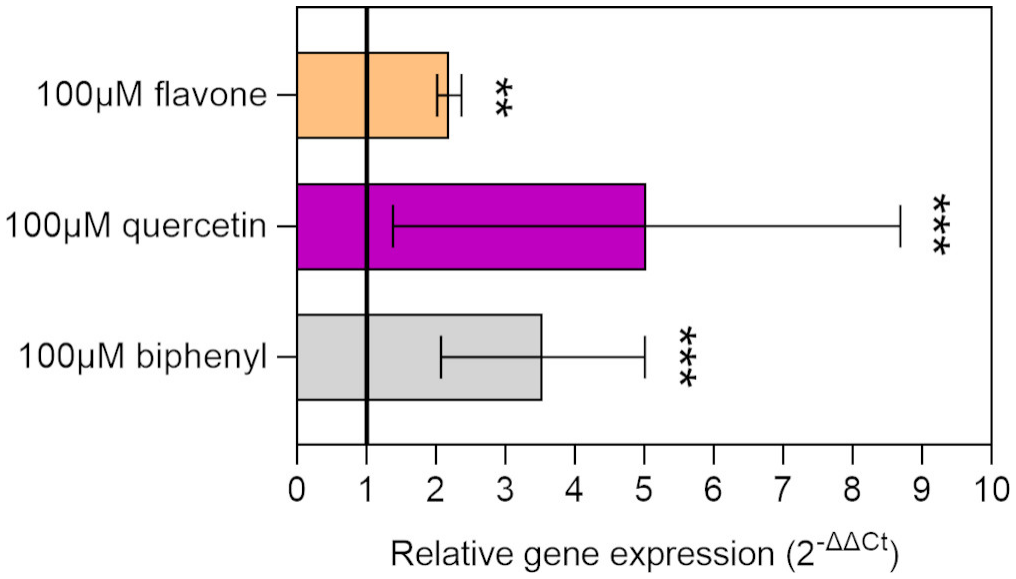
Relative gene expression of the *bphA* gene in strain LB400 exposed to flavonoids. The relative gene expression of the *bphA* gene was quantified via RT-qPCR, using the 16S rRNA gene as a housekeeping gene. The results of the relative expression of *bphA* transcripts are expressed as ΔΔCt, indicating the average fold change over the non-induced control. The black line (relative gene expression value = 1) represents the baseline of gene expression in the non-induced control. Biphenyl was used as positive control. Error bars represent the standard deviation of three independent experiments. Statistical analysis was performed using Dunn’s *post-hoc* test and asterisks indicate significant differences from the control (**p ≤ 0.01, ***p ≤ 0.001).

Flavonoids were demonstrated to influence the PCB degradative potential of soil microorganisms, acting as inducers or co-metabolites of the catabolic operon *bph* (Toussaint et al., 2012; Zubrova et al., 2021). The ability of individual flavonoid molecules to induce the transcription of the degradative machinery in strain LB400 was assessed by monitoring *bphA* gene relative expression by RT-qPCR. The relative induction rates indicated that two of the assayed flavonoids, namely, flavone and quercetin, were able to increase the levels of *bphA* transcripts after 8 h of incubation, similarly to biphenyl, the model inducer of the *bph* operon (**Figure 3**). These observations highlight flavonoid ability to activate the expression of PCB degradative traits in *P. xenovorans* LB400.

### 3.4 Differential exudation of flavonoids affects *P. xenovorans* LB400 early adhesion on *Arabidopsis* roots

**Figure 4:**
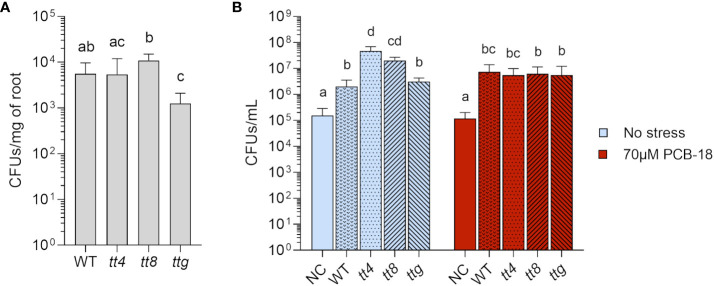
Root exudates from *Arabidopsis* mutants with distinct flavonoids exudation pattern affected *P. xenovorans* LB400 early colonization and were diversely exploited by the bacterium for growth. **(A)** Early colonization was measured via re-isolation of strain LB400 from *Arabidopsis* roots after 1 h of incubation. The graph reported the average and standard deviation values of three independent replicates for all the assayed *Arabidopsis* genotypes. Statistical analysis was performed using Dunn’s *post-hoc* test, and letters indicate statistically different groups (p ≤ 0.05). **(B)** Strain LB400 growth on root exudates collected from WT *Arabidopsis* and flavonoid metabolic mutant lines at day 7 of treatment under control conditions (plants exposed to acetone, the solvent used to dissolve PCB-18) or PCB stress induced by the treatment with 70 µM PCB-18. NC indicates 1/2 MS medium containing only acetone or 70 µM PCB-18, without plants, and was used as axenic control. Strain LB400 was inoculated at a concentration of 5 × 10^4^ cells/mL and grown for 3 days before re-isolation. The bars represent the average and standard deviation values of three independent experiments. Statistical analysis was performed using Dunn’s *post-hoc* test and letters indicate statistically different groups (p ≤ 0.05).

Given the results obtained with flavonoids as pure chemicals, we verified if differences in the amount and chemistry of flavonoids in the complex natural blend of exuded compounds released by plant roots could affect strain LB400 growth and root adhesion efficiency. For this purpose, the *Arabidopsis* mutant lines *tt4*, *tt8*, and *ttg*, differentially altered in flavonoid biosynthesis and exudation (Narasimhan et al., 2003), were adopted. To investigate the effect of root exudates on strain LB400 root adhesion ability, *Arabidopsis* plantlets of the different genetic backgrounds were exposed for 1 h to the bacterial culture before proceeding with the re-isolation of cells adhering on the rhizoplane, aiming to focus on the role of flavonoids in the early events of bacterial colonization. It was observed that in 1 h, strain LB400 colonized both WT and *tt4* roots at a similar rate (5.57 × 10^3^ and 5.42 × 10^3^ CFUs/mg of root fresh weight, respectively) whereas the bacterial density increased in the *tt8* line, which accumulates flavonoid aglycones (1.09 × 10^4^ CFUs/mg of root fresh weight) (**Figure 4A**). On the contrary, roots of *ttg*, which overproduces flavonoid conjugates, were less colonized compared with the other lines (1.25 × 10^3^ CFUs/mg of fresh root weight) (**Figure 4A**). These results suggest that two distinct flavonoid-overexpressing fingerprints differently affect strain LB400 colonization ability in the early events of root adhesion.

### 3.5 Flavonoid exudation affected the bacterial ability to exploit plant root exudates as nutrient sources

We tested whether the complex blend of root exudates produced by the mutants could sustain bacterial growth as a nutrient source and eventually provide signaling molecules for enhanced bacterial proliferation. WT and *ttg* mutant plant exudates showed a similar ability to support strain LB400 growth (**Figure 4B**, light blue bars). On the other hand, the exudates released by *tt4* and *tt8* relevantly contributed to enhance bacterial growth, allowing the formation of a higher bacterial biomass (4.66 × 10^7^ CFUs/mL and 2.02 × 10^7^ CFUs/mL for *tt4* and *tt8* lines, respectively).

Considering flavonoid involvement in the expression of the *bphA* gene (**Figure 3**), root exudates were also collected from plantlets subjected to PCB-18 stress, to assess if strain LB400 could be a target for a potential “cry-for-help” strategy. The exudates collected from the WT and the different *Arabidopsis* mutants cultivated in presence of 70 µM PCB-18 showed the capacity to sustain strain bacterial growth (**Figure 4B**, red bars). Strain LB400 growth was unaffected by the presence of stress-triggered exudates released by WT and *ttg*, showing no major differences in using the root exudates released by these lines in the presence and absence of PCB stress. On the other hand, strain LB400 growth dropped of one order of magnitude with *tt4* exudates released upon PCB-18 stress (5.49 × 10^6^ CFUs/mL) compared with the control conditions, and similarly, a decrease was also observed in the bacterial ability to exploit *tt8* exudates released under stress compared with the control.

### 3.6 *P. xenovorans* LB400-mediated plant growth promotion under control conditions and under PCB stress showed a similar pattern in *Arabidopsis* lines affected in flavonoid biosynthesis

**Figure 5:**
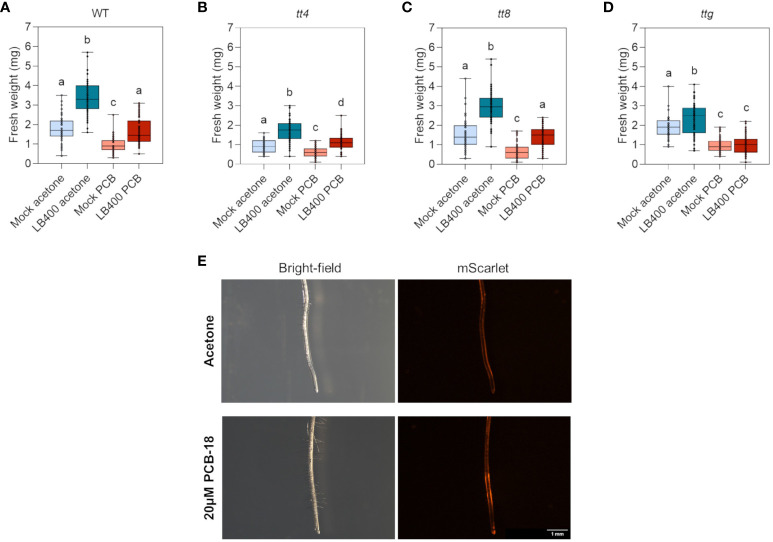
*P. xenovorans* LB400 growth promotion, colonization efficiency, and root adhesion profile were not differentially affected in *Arabidopsis* plants with diverse profiles of flavonoid biosynthesis and exudation. **(A–D)** Root fresh biomass of sterile and strain LB400-colonized *Arabidopsis* plantlets grown under control conditions (acetone) and challenged by 20 µM PCB-18 treatment in WT **(A)**, *tt4* **(B)**, *tt8* **(C)**, and *ttg* **(D)** lines, respectively. The boxplots represent data from three independent experiments. Letters indicate statistically different groups (Dunn’s *post-hoc* test with p ≤ 0.05). **(E)** Fluorescence microscopy analysis of the *mScarlet*-labelled LB400 strain colonization pattern on the root system of WT plantlets 7 days after PCB treatment or in control conditions (acetone).

The role of flavonoids in strain LB400–plant interaction was investigated *in planta* by taking advantage of a well-established microcosm system set up to assess the interaction between beneficial microbes and *Arabidopsis* plantlets (de Zélicourt et al., 2018; Rolli et al., 2022). In addition, a specific in-plate assay was developed in this study to simulate PCB-induced phytotoxicity in *Arabidopsis*. It was observed that the exposition to 20 µM PCB-18 dramatically affected plant growth and development and prompted a series of injuries attributable to PCB stress (**Supplementary Figure 4**). Indeed, when grown for 14 days in the presence of PCB-18, all the assayed *Arabidopsis* mutant lines reported a significant reduction in root fresh weight that was coupled also to a decrease in total plant biomass, depending on the genotypes (**Figures 5A–D** and **Supplementary Figure 5**). By analyzing root architecture, only slight differences were observed in terms of primary root length and number of secondary roots, suggesting that PCB-18 stress mainly affected root biomass rather than its morpho-phenotypic traits (**Supplementary Figure 6**). This assay was adopted to compare strain LB400-mediated growth promotion in the WT plants with *tt4*, *tt8*, and *ttg* mutant lines under control conditions and in the presence of PCB stress. Colonization with strain LB400 promoted plant growth by increasing plant biomass of both shoots and roots in WT and all mutant plant lines grown under control conditions (**Figures 5A–D** and **Supplementary Figure 5**). In particular, in the root system, strain LB400 induced the formation of a longer primary root, whereas the number of secondary roots showed a variation that was genotype-related (**Supplementary Figure 6**). These findings underlined that strain LB400 possesses plant growth promoting (PGP) traits and that the promotion activity was not altered in *Arabidopsis* backgrounds that are featured by null synthesis or overproduction of flavonoids. The PGP potential of LB400 was further investigated through quantitative *in vitro* tests, demonstrating that *P. xenovorans* LB400 possesses a large portfolio of PGP traits, including auxin synthesis (31.4 µg IAA/OD_600_), ACC-deaminase activity (32.7 µg αKB/OD_600_), EPS production (2.08 µg bound Congo red/OD_600_), siderophore release, and production of VOCs (**Supplementary Figure 7**). These beneficial features, linked with the PCB degradative activity of the strain, may explain strain LB400 ability to sustain plant growth both under control conditions and under PCB-18 stress. Remarkably, LB400 demonstrated to mitigate the toxicity stress in plants cultivated in the presence of 20 µM PCB-18 for WT and the *tt4* and *tt8* mutants whereas only the *ttg* mutant did not show the beneficial effect provided by the bacterial inoculant (**Figures 5A–D** and **Supplementary Figure 5**). No major changes in *Arabidopsis* colonization pattern were observed either by re-isolation (**Supplementary Figure 8**) and by fluorescence microscopy analysis (**Figure 5E** and **Supplementary Figures 9** and **10**).

## 4 Discussion

Root chemistry alteration by the components of the holobiont, either the host plant or the associated microbiota, is emerging as a valuable strategy for the recruitment and the preservation of the microbial functionalities that result in the consolidation of the holobiont fitness under stress, guaranteeing its useful services like rhizoremediation (Voges et al., 2019; Korenblum et al., 2020). The pillar of this process is the structural affinity between the contaminants and plant secondary metabolites that drive their degradation through the same enzymatic machinery of the microbial peripheral pathway. In this vision, the ecological services provided by the *bph* operon are hijacked from the degradation of biphenyl-like PSMs to also allow PCB catabolism, conferring a selective advantage to *bph*-equipped bacteria in polluted rhizosphere soil. Therefore, bioremediation relies on evolutionary-shaped processes, comprising the enzymatic versatility of microbial metabolism and the inter-kingdom trophic interactions among plants and microbes.

Among others, flavonoids constitute a broad group of specialized secondary metabolites (Vives-Peris et al., 2020), consistently abundant in the root exudates of several plant species including legumes (Leoni et al., 2021) and trees like *Acer saccharum*, *Alnus rugosa*, *Fagus grandifolia*, *Picea abies*, *Pinus strobus*, and *Quercus rubra* where catechin, naringenin, and taxifolin represented the most abundant exudates (Zwetsloot et al., 2018). Recent evidence supports the vision of a broader role for flavonoids in plant–microbe dynamics that could allow the development of beneficial relationships also in non-nitrogen-fixing crops and with non-rhizobial bacterial species (Yu et al., 2020; Kudjordjie et al., 2021; Yu et al., 2021; He et al., 2022). In this framework, our aim was to investigate whether flavonoids could affect the rhizocompetence and the degradation potential of the model PCB-degrader *P. xenovorans* LB400, thus contributing to the recruitment of a beneficial strain able to alleviate plants from the phytotoxic damages caused by these recalcitrant compounds (Rolli et al., 2021).

Our findings indicate that the exposure of strain LB400 to micromolar concentrations of plant flavonoids, supplied as pure chemicals, can selectively modulate the growth of the bacterium, affecting its proliferation, biomass yield, and maximum growth rate, both in relation to the specific flavonoid molecule type and its concentration. Similarly, some triterpene compounds like thalianin and arabidin previously demonstrated to specifically affect members of *Arabidopsis* root microbiota by promoting or inhibiting their growth and by serving as carbon sources (Huang et al., 2019). This flavonoid-triggered effect could drive relevant consequences in the proliferation ability of degrading bacteria in the rhizosphere of plants growing in PCB contaminated environments. Although considered the largest biome on earth, soil is an oligotrophic environment subjected to fluctuations in nutrient and water availability (Hartmann and Six, 2023). Endowed with a 15-carbon skeleton, flavonoids represent valuable nutrient sources for those rhizosphere microorganisms harboring appropriate catabolic enzymes, like *Rhizobium*, *Bradyrhizobium*, *Acinetobacter*, and *Pseudomonas* (Rao and Cooper, 1995; Pillai and Swarup, 2002; Arunachalam et al., 2003). *Pseudomonas putida* PLM2 showed to aerobically catabolize quercetin through oxidation and/or reduction reactions that led to the formation of catechol (Pillai and Swarup, 2002), and *P. xenovorans* LB400 itself was able to use morusin, morusinol, and kuwanon C, flavones produced by fine roots of mulberry, as sole carbon sources (Leigh et al., 2002). Therefore, the ability to exploit the flavonoid resource may represent an important selective value in plant–microbe interactions. It has been shown that the chemical variation of *Avena barbata* root exudates along the plant lifespan is coupled to the microbial succession of different phyla that encode for uptake systems and catabolic enzymes useful to exploit exudates as nutrient sources (Zhalnina et al., 2018). Indeed, micromolar supply of quercetin to an agricultural soil differentially affected specific microbial taxa, increasing up to 76% the relative abundance of Proteobacteria (Schütz et al., 2021). In agreement with our findings on flavonoid-triggered growth promotion in *P. xenovorans* strain LB400, *Bradyrhizobium* strain ORS285 can metabolize naringenin into a hydroxylated and methoxylated derivative that did not act as a Nod inducer. Instead, it stimulated bacterial growth presumably by regulating the glycerol and fatty acid metabolism (Nouwen et al., 2019). These results suggest that the assayed flavonoid compounds, being used as carbon sources or proliferation-stimulating agents, may contribute to fuel microbial growth and to increase *P. xenovorans* LB400 proliferation and persistence in the rhizosphere.

Furthermore, our results demonstrated that naringin and naringenin enhance swimming motility, chemotaxis, and biofilm formation in strain LB400, traits that are often enriched in the genomes of plant-associated bacteria compared with soil-inhabiting ones (Levy et al., 2018) and that are believed to be involved in rhizosphere recruitment and colonization (Santoyo et al., 2021). Our observations support the evidence that root-secreted flavonoids constitute a carbon-rich reservoir that might induce priming mechanisms in soil microorganisms (Zwetsloot et al., 2018). This function is not necessarily related to the use of flavonoids as primary carbon sources but also to their role as intermediate substrates, signaling molecules, or attractants that can stimulate various metabolic activities, useful to support plant growth and survival in harsh conditions. Indeed, most of the bacteria isolated from the rhizosphere of maize, mustard cabbage, and lettuce grew similarly well on both host and non-host exudates, indicating a large metabolic versatility to exploit plant released compounds (Dhungana et al., 2023).

Studying the temporal dynamics of root exudation, McLaughlin and coauthors demonstrated that in few hours the main determinants of three distinct plant species exudate patterns were released by the roots and their concentration then increased with time (McLaughlin et al., 2023). This evidence motivated the use in this work of three *Arabidopsis* lines that are affected in flavonoid biosynthesis to verify whether strain LB400 was differentially recruited in the rhizoplane. *Arabidopsis thaliana* stands as a proficient model for holobiont dynamics under abiotic stresses (Poupin et al., 2023) and offers a large portfolio of well-characterized genetic and metabolic resources, especially for flavonoid biosynthesis, regulation, and exudation (Nakayama et al., 2019). It was observed that in 1 h, the WT and the null-producer *tt4* line were similarly colonized, *tt8* roots were colonized at higher efficiency whereas *ttg* roots were far less colonized. In the assayed mutant lines, flavonoids are the root exudates mainly affected and therefore they vary largely in quantity and composition. Nevertheless, in the complex blend of exudates released by these mutant lines, also other molecules of the phenylpropanoid pathway result to be differentially exuded (Narasimhan et al., 2003), thus making it intricate to distinguish an effect mediated undoubtedly by flavonoids. The scenario is even more complex, considering that different flavonoids have been showed to exert contrasting functions in the recruitment of microbial taxa in the rhizosphere (Wang et al., 2022). Indeed, naringenin strongly improved *Aeromonas* sp. chemotaxis and motility, potentially explaining the higher colonization rate in *pap1-D* mutant that overaccumulates anthocyanins and flavonols (He et al., 2022). On the other hand, daidzein, that possesses antimicrobial activity, decreased the α-diversity of the microbial community in soybean rhizosphere, suggesting a potential role as chemorepellent (Okutani et al., 2020). Tadra-Sfeir and colleagues reported a transcriptomic study on the diazotrophic bacterium *Herbaspirillum seropedicae*, highlighting that naringenin repressed bacterial motility while enhancing early root colonization and activating the expression of genes related to the degradation of aromatic compounds (Tadra-Sfeir et al., 2015). Similarly, we observed that different flavonoid molecules can exert specific effects: while quercetin and flavone did not contribute to bacterial swimming motility, they acted as inducers of the PCB degradative pathway. Some of the observed effects are also concentration-dependent, as in the case of flavone-mediated promotion of strain LB400 growth. No precise information is available about flavonoid concentration in the rhizosphere, also considering the impact of abiotic processes like adsorption to soil particles or biological degradation by the soil microbiota (Shaw et al., 2006; Del Valle et al., 2020). Therefore, there is the possibility that flavonoid concentrations used for the *in vitro* experimental setup are higher than those available in the rhizosphere. In axenic systems for root exudate collection, for instance, flavonoids were estimated in micromolar ranges (Toussaint et al., 2012), suggesting that the concentrations used in the present study can indeed affect bacterial physiology and plant–bacteria interactions mimicking the real conditions that could be found in soil.

Our interest in testing the selected *Arabidopsis* lines was also coupled to their previous exploitation in a rhizo-engineering strategy for PCB removal by the degrading strain *Pseudomonas putida* PML2 (Narasimhan et al., 2003). Importantly, all the different blends of exudates released by the *Arabidopsis* lines used in this study could successfully support *P. xenovorans* growth, independently from their over- or underproduction of flavonoids given by the genetic mutation. Presumably, some of the aromatic organic acids that are overaccumulated in the absence of flavonoids in *tt4* exudation, like cinnamic acid and indole-3-acetic acid (Narasimhan et al., 2003), which were previously highlighted as preferentially consumed by rhizospheric microorganisms (Zhalnina et al., 2018), could contribute to the enhanced ability of sustaining strain LB400 growth under control conditions. The changes in the exudation pattern induced by the “cry-for-help” in the presence of PCB stress are still unknown. Here, we observed that no major changes occurred in strain LB400 growth in the presence of exudates from WT plants cultivated with or without PCB, thus excluding a prompt “cry-for-help” effect, as already observed for *Aeromonas* sp. H1 under dehydration stress (He et al., 2022). In any case, the growth of strain LB400 on exudates released under PCB-18 stress decreased for *tt4* and *tt8* mutants, exhibiting absent (*tt4*) or modified flavonoid biosynthesis (*tt8*), thus potentially supporting a role of flavonoids and/or their aglycones in nurturing degrading bacteria. Bacterial growth was not affected in the presence of the flavonoid-hyperproducing *ttg* mutant exudates, independently from the treatment. This mutant line releases conjugated flavonoids, which are more hydrophilic, mobile, and bioavailable molecules. Therefore, they are considered short-lived forms that can be potentially rapidly degraded by plant and microbial glucosidases (Hartwig et al., 1991), leading to formation of byproducts that are still poorly characterized for their effect on soil microbiota and influence in the rhizosphere dynamics (Shaw et al., 2006).

Based on previous research on PCB-18 toxicity in *Arabidopsis* (Bao et al., 2013), an *in vitro* assay was developed that simulated the phytotoxic effects induced by PCBs that led to reduced plant growth and development, with the aim to assess *P. xenovorans* LB400–plant interaction under PCB stress. As a model strain for PCB removal (Chain et al., 2006), strain LB400 was previously used mainly in bioaugmentation approaches for contaminated sediments (Tehrani et al., 2014; Le et al., 2015; Bako et al., 2022), whereas its association with plant roots is still largely overlooked. Recently, *Paraburkholderia* phylotypes, matching with *P. xenovorans* LB400, were retrieved in forest soil with sugar maple, red pine, and black locust trees as the dominant p-hydroxybenzoic acid responder, supporting the notion of the presence of a specialized metabolism to degrade phenolics upon priming (Wilhelm et al., 2021). Therefore, the present study contributes to widen the knowledge on *P. xenovorans* LB400 beneficial contribution to plant growth showing that, in addition to its well-documented PCB degradation ability (Ponce et al., 2011), this bacterial strain is also endowed with a large portfolio of PGP traits, potentially contributing to further boost plant fitness under stress (Marasco et al., 2012; Vigani et al., 2019). Strain LB400 administration to the plant contributed to improve both shoot and root biomass, with the root weight being the growth parameter that was mostly affected by PCB-18 stress in the developed assay. *P. xenovorans* LB400 exerted beneficial effects both under control conditions and under stress, in WT and *Arabidopsis* mutants with null (*tt4*) or overexpressing (*tt8*) flavonoid metabolism, whereas it did not show to improve the growth of the overproducer *ttg* mutant line when growing under PCB stress. Considering also that the degrading strain *Pseudomonas putida* PML2 performed the most remarkable PCB depletion (more than 90%) in the WT *Arabidopsis* roots rather than in the mutants (Narasimhan et al., 2003), these observations may suggest that the bacterial-driven plant growth promotion under control conditions and under PCB stress could be achieved through a flavonoid-independent mechanism.

Our results delineate a scenario in which these secondary metabolites play a pivotal role in facilitating the early bacterial recruitment in the rhizoplane. Indeed, once the bacterium is settled on the root surface, strain LB400 colonization efficiency (at 7 and 14 DAT) and adhesion pattern (at 7 DAT) did not differ among the investigated *Arabidopsis* genotypes. A similar trend was also observed for *P*. *putida* PML2, which colonized with a similar efficiency the three *Arabidopsis* lines that produce flavonoids, although the *tt4* line presented less bacterial cells (Narasimhan et al., 2003).

Remarkably, a fluorescence-labeled version of strain LB400 was developed within this study, contributing to specifically visualize the colonization profile on the root system. It was observed that strain LB400 established homogeneously on the primary roots from the differentiation zone to the apical region, whereas secondary roots were not colonized 7 DAT. Nevertheless, it was previously observed that *Bacillus subtilis* established early in the root elongation zone of *Arabidopsis*, a potential hotspot for exudation, but this event preceded the colonization over the entire root length (Knights et al., 2021). Interestingly, this colonization pattern complied with the bacterial-mediated remodeling of root architecture that mainly affected primary root length and biomass. Often, bacterial-triggered remodeling of root architecture is mediated by the alteration of plant hormone homeostasis. The PGP strain *Achromobacter* sp. 5B1, for instance, influenced the growth and branching pattern of *Arabidopsis* roots through a redistribution of auxins in the primary roots, with the result of improving salt resistance (Jiménez-Vázquez et al., 2020). Strain LB400 was shown to be able to produce IAA and VOCs and express the ACC-deaminase enzyme, having thus the potential to interfere with the plant hormone homeostasis.

In addition to PGP traits, bacterial degradative ability is an essential beneficial factor in plant–microbe interaction in contaminated soils. *P. xenovorans* LB400 is a well-known PCB degrader, and our study contributes to further explore its degradation potential by demonstrating the ability of flavone and quercetin to activate the transcription of the *bph* operon at similar levels as biphenyl. Although possessing genetic differences (Hirose et al., 2019), isoflavone and quercetin, among other flavonoids, acted as co-metabolites by inducing the *bph* operon expression in the degrading strains *Rhodococcus erythropolis* U23A and *Pseudomonas alcaliphila* JAB1 (Pham et al., 2015; Zubrova et al., 2021).

To summarize, our results are in line with recent evidence claiming a broader role for flavonoids in the recruitment and assembly of the plant microbiota with regard to non-rhizobial strains (He et al., 2022). This role includes the early sustainment of bacteria in the rhizoplane in two ways: (i) recruitment by stimulating chemotaxis and motility processes, resulting in root attachment through biofilm formation and (ii) induction of proliferation of the bacteria established on the root by their action as nutrients or growth stimulator signals. Furthermore, flavonoids improve strain LB400 degradative functionalities for PCB removal, with the potential effect of reducing soil phytotoxicity. These aspects are crucial in rhizo-remediation strategies, whose success rely in the synergistic activities performed by the host plant and the degrading microbiota. Nevertheless, our results specifically highlight that flavonoids are important to initiate *Arabidopsis* root colonization, but once a baseline colonizing community is established, at longer time intervals, presumably other mechanisms and/or other plant exuded metabolites occur in plant–bacteria interaction to sustain bacterial growth and colonization pattern, both under control conditions and PCB stress.

More research is needed to disclose the differential composition of root exudation under PCB stress and identify the key secondary metabolites that are released by the plant following the “cry-for-help”. Although single-strain studies in axenic plant systems contribute to determine the causative mechanisms, exploiting PCB-degrading synthetic communities would be useful to address the complexity of interactions among bacteria and host plants in contaminated environments. With this knowledge, it would be possible to mechanistically demonstrate the impact of stress-triggered root exudation in recruitment, colonization, and functional features of degrading bacteria, with the ultimate goal to exploit them to sustain rhizoremediation services in contaminated soils.

In the perspective of a field approach, the use of plant secondary metabolites as specific biostimulants to shape the microbiome toward desired services is an attractive strategy for rhizoremediation (Koprivova and Kopriva, 2022). Such approach was applied to facilitate the mineralization of organic phosphorus, showing that soil amendments with quercetin, naringenin, or luteolin increased the relative abundance of *Micrococcaceae* and *Nocardioidaceae*, bacterial families that positively correlated with enhanced alkaline phosphatase activity, in rhizospheric soil (Wang et al., 2023). A major limitation to this kind of approach is the reduced lifetime of flavonoids in soil, estimated to decrease up to 63%, due to sorption or other reactions with dissolved organic carbon (Del Valle et al., 2020). On the other side, foliar application of rutin significantly enhanced *Amaranthus hypochondriacus* phytoremediation efficiency to remove cadmium by favoring its immobilization in the cell wall rather than in the vacuole (Kang et al., 2022).

The other crucial players in rhizoremediation are plants: so far, species showing resilience, high biomass production, resistance to pollutants’ toxic effects, and easy-to-grow ability have been identified (Kafle et al., 2022). This is the case of *Festuca arundinacea* that was broadly applied for land reclamation of soils exhibiting a diverse array of pollution profiles, including hydrocarbons, heavy metals (Khashij et al., 2018), and PCBs as well (Terzaghi et al., 2019). Improving this knowledge would allow, for instance, the selection of plants exhibiting specific host phenotypes, including an enriched exudation of specific metabolites, to select for more efficient PCB degrading strains through host-mediated microbiome engineering.

## Data availability statement

The datasets presented in this study can be found in online repositories. The names of the repository/repositories and accession number(s) can be found below: UNIMI Dataverse repository at https://dataverse.unimi.it/dataverse/SENSE.

## Author contributions

EG: Conceptualization, Data curation, Formal analysis, Investigation, Methodology, Validation, Visualization, Writing – original draft, Writing – review & editing. ER: Conceptualization, Data curation, Formal analysis, Funding acquisition, Investigation, Methodology, Project administration, Supervision, Validation, Visualization, Writing – original draft, Writing – review & editing. LV: Investigation, Writing – original draft, Writing – review & editing. SB: Conceptualization, Funding acquisition, Project administration, Resources, Supervision, Writing – original draft, Writing – review & editing.
