# Supplementary material for: Flavonoids influence key rhizocompetence traits for early root colonization and PCB degradation potential of Paraburkholderia xenovorans LB400
Source: Front Plant Sci. 2024 Feb 2;15:1325048. doi: 10.3389/fpls.2024.1325048 (PMC10869545; doi:10.3389/fpls.2024.1325048)
Supplement: Supplementary file 1 [file DataSheet_1.pdf]

Supplementary Material

Flavonoids influence key rhizocompetence traits for early root colonization and PCB degradation potential of *Paraburkholderia xenovorans* LB400

Elisa Ghitti[1], Eleonora Rolli[1*], Lorenzo Vergani[1], Sara Borin[1]

1. Department of Food, Environmental and Nutritional Sciences (DeFENS), University of Milan, Milan, Italy.

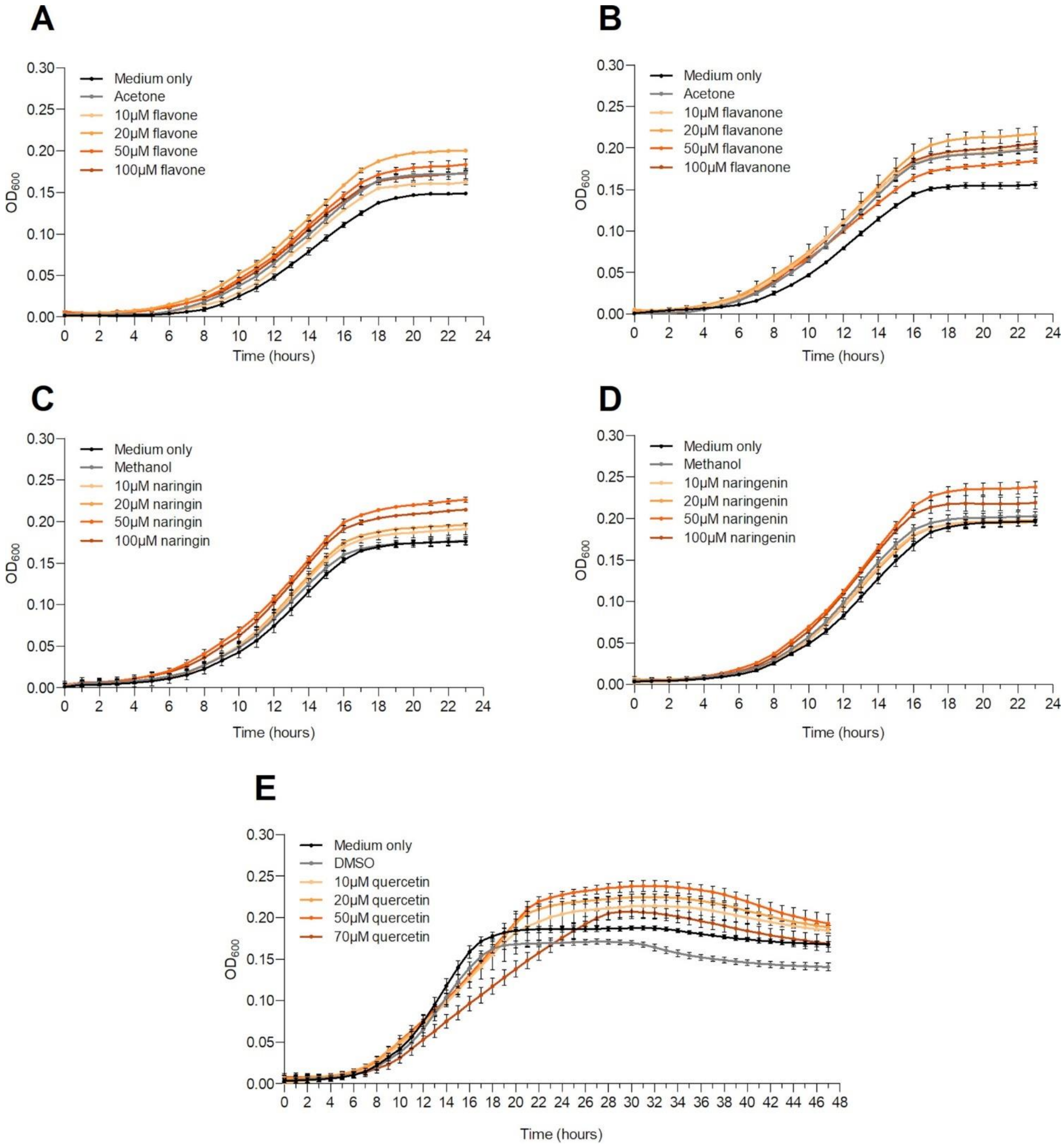

Supplementary Figure 1. Growth curves of the strain *P. xenovorans* LB400 in presence of flavonoids. The bacterium was assayed for growth in 1/10 TSB medium supplemented with the following flavonoids: (**A**) flavone, (**B**) flavanone, (**C**) naringin, (**D**) naringenin and (**E**) quercetin at final concentrations of 10, 20, 50, 70 and 100 µM. "Medium only" indicates growth in presence of 1/10 TSB with no additions, while "acetone", "methanol" and "DMSO" indicate growth in 1/10 TSB plus the solvents in which flavonoids were dissolved. The average and standard deviation values of three biological replicates are reported for each treatment and for each time point.

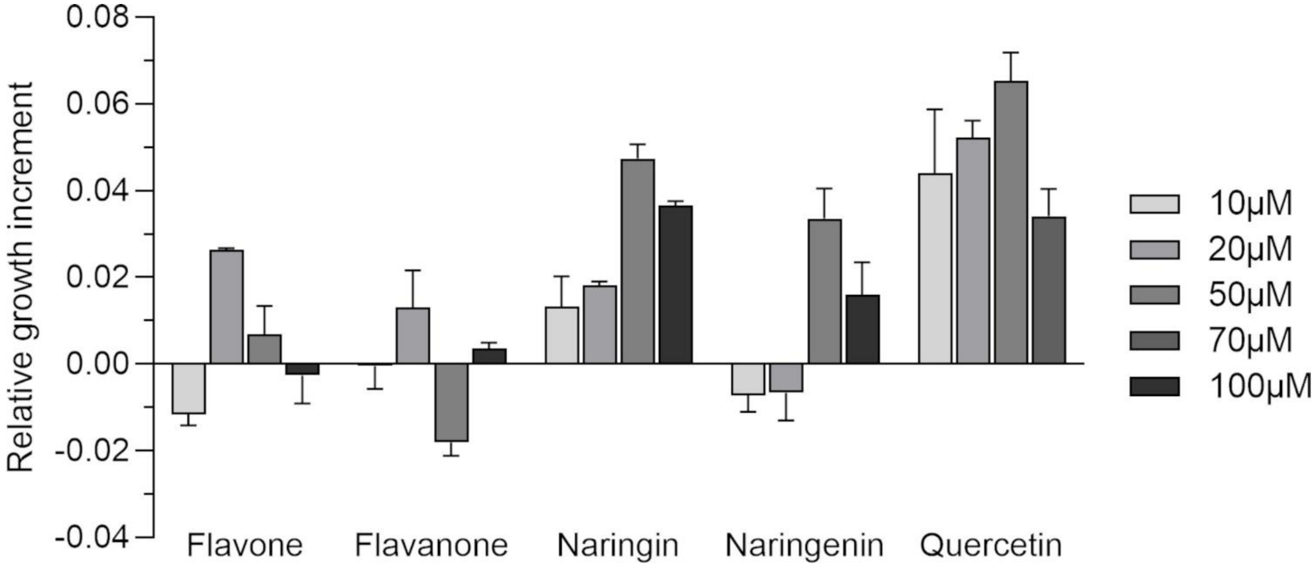

Supplementary Figure 2. Relative growth increment of strain LB400 at stationary phase. The relative growth increment was calculated as the ratio of the bacterial growth in 1/10 TSB medium supplemented with different concentrations of flavonoids (10, 20, 50, 70 and 100 μM) and the growth in presence of the respective solvents in which flavonoids were dissolved. The average and standard deviation values of three biological replicates are reported for each treatment.

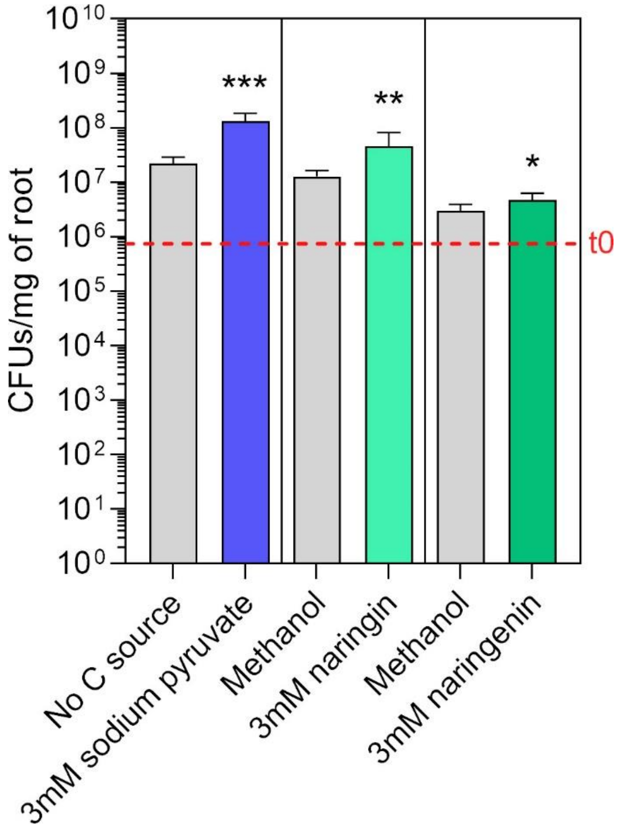

Supplementary Figure 3. *P. xenovorans* LB400 uses the flavonoids naringin and naringenin as carbon sources. The bacterium was grown for 6 days in mineral medium supplemented with either 3 mM sodium pyruvate as positive control or 3 mM naringin and naringenin. A corresponding amount of the respective solvent was used as control. The dashed red line represents the cell density that was inoculated in each vial at the beginning of the experiment (t0). Average and standard deviation values of two biological replicates, each with three technical replicates, are reported for each treatment. Statistical analysis was performed using the Mann-Whitney test (*: $p \leq 0.05$, **: $p \leq 0.01$, ***: $p \leq 0.001$).

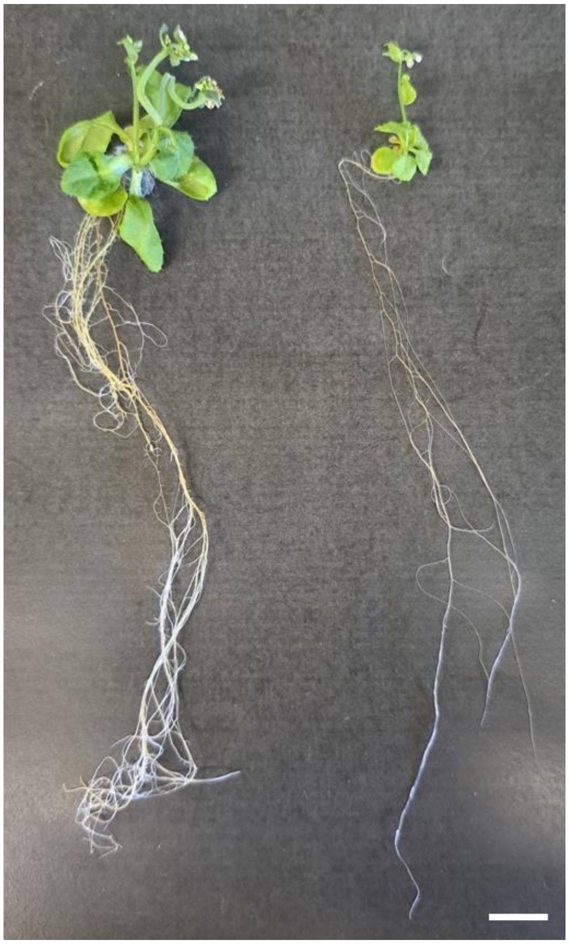

Supplementary Figure 4. *In vitro* PCB-18-induced stress in *Arabidopsis thaliana* wild type (WT) L*er* ecotype. In the present work, an *in vitro* PCB-18-induced stress experiment was set-up as described in the material and methods section. In the image, two representative plants showing the effects on *Arabidopsis* growth in presence of 20 µM PCB-18. In this case, the plantlets were deliberately grown for 28 days, a longer period compared the time points analyzed in this study (7 and 14 days) to clearly observe the PCB-18-induced phytotoxic effect on plant growth and development. PCB-18 phytotoxic effect causes a reduction in shoot and root biomass (plant on the right) if compared with a plant grown under control conditions in 1/2 MS supplemented with acetone as untreated control (plant on the left). Size of the bar: 1 cm.

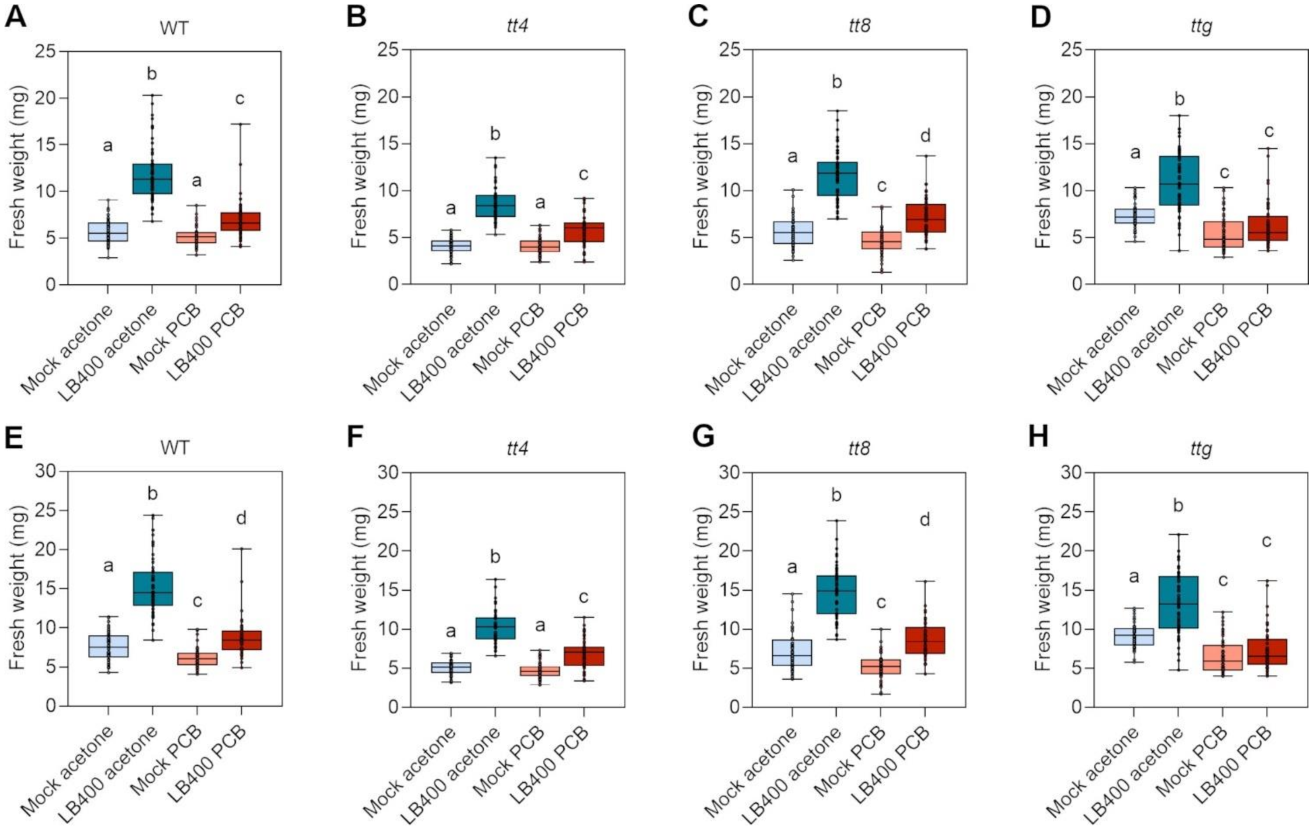

Supplementary Figure 5. PCB-18-induced decrease in plant fresh weight in WT plants and *Arabidopsis* lines affected in flavonoid biosynthesis and exudation. (**A**), (**B**), (**C**) and (**D**): shoot fresh weight in axenic (mock) or *P. xenovorans* LB400-colonized plants grown under control conditions (acetone) or exposed to PCB-18 in WT, *tt4*, *tt8* and *ttg* genotypes. (**E**), (**F**), (**G**) and (**H**): total plant fresh weight in axenic (mock) or *P. xenovorans* LB400-colonized plants grown under control conditions (acetone) or exposed to PCB-18 in WT, *tt4*, *tt8* and *ttg* genotypes. The boxplots represent data from three independent experiments. Letters indicate statistically different groups (Dunn's post-hoc test with $p \leq 0.05$).

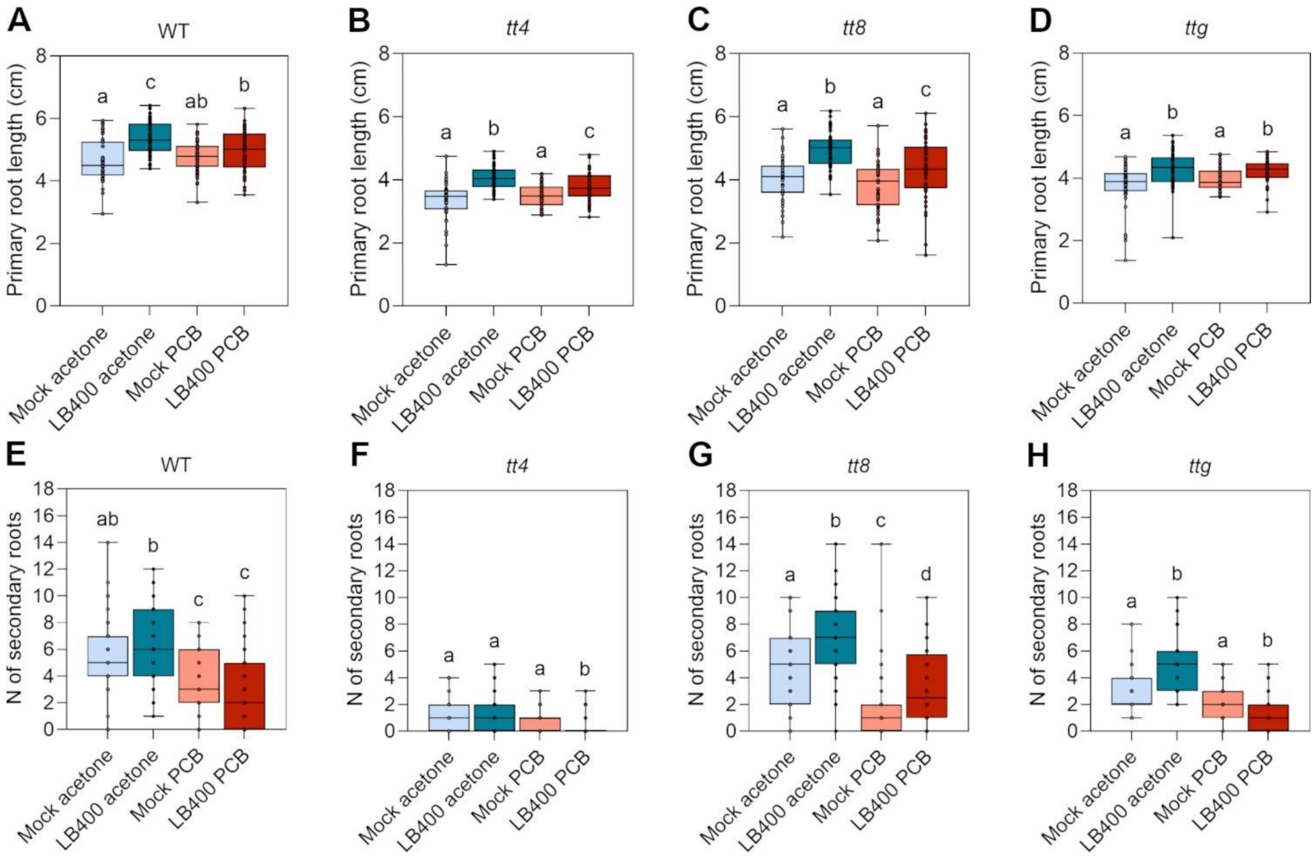

Supplementary Figure 6. PCB-18-induced remodeling of root architecture traits in WT plants and *Arabidopsis* lines affected in flavonoid biosynthesis and exudation. (**A**), (**B**), (**C**) and (**D**): primary root length in axenic (mock) or *P. xenovorans* LB400-colonized plants grown under control conditions (acetone) or exposed to PCB-18 in WT, *tt4*, *tt8* and *ttg* genotypes. (**E**), (**F**), (**G**) and (**H**): number of secondary roots in axenic (mock) or *P. xenovorans* LB400-colonized plants grown under control conditions (acetone) or exposed to PCB-18 in WT, *tt4*, *tt8* and *ttg* genotypes. The boxplots represent data from three independent experiments. Letters indicate statistically different groups (Dunn's post-hoc test with $p \leq 0.05$).

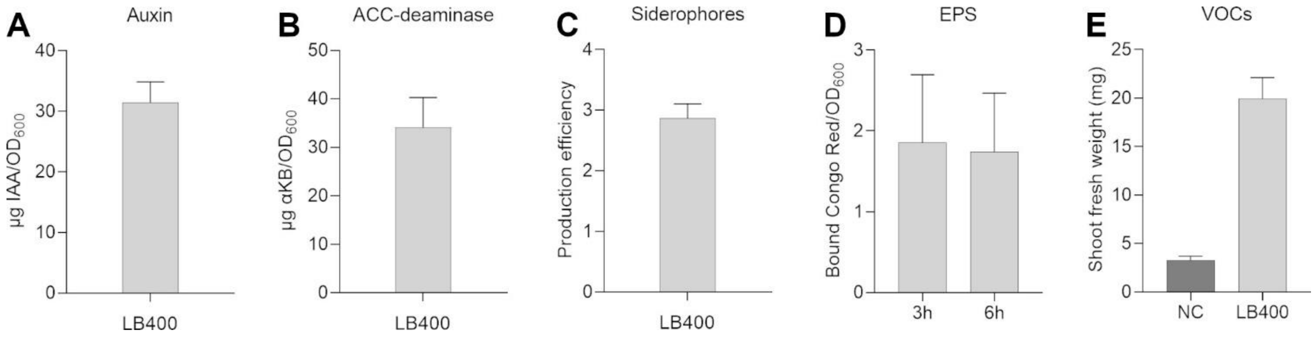

Supplementary Figure 7. *P. xenovorans* LB400 exhibited multiple plant growth promoting traits *in vitro*. The bacterium was assayed for auxin biosynthesis (**A**), expression of ACC-deaminase activity (**B**), siderophore release (**C**), EPS production (**D**) and VOCs release (**E**).

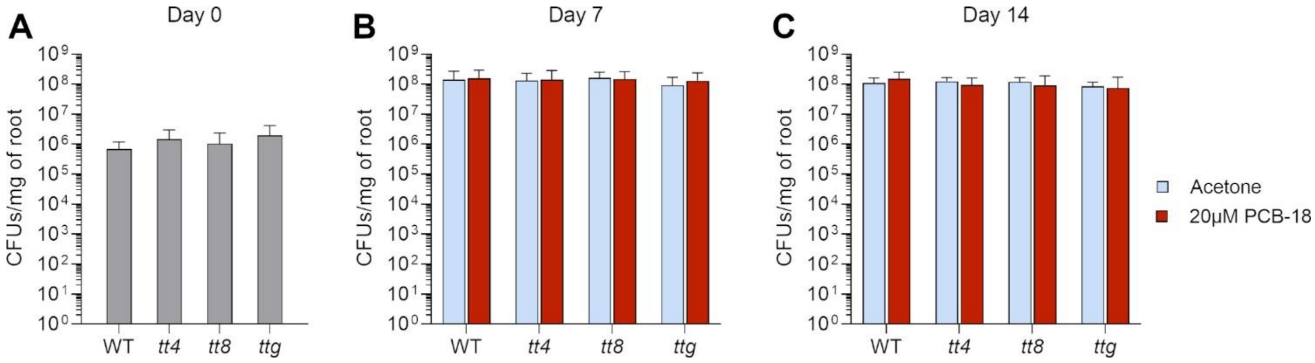

Supplementary Figure 8. Strain LB400 colonization efficiency in *Arabidopsis* plantlets at day 0, day 7 and day 14 after transfer on PCB-stressed plates in the *in vitro* assay to evaluate PCB-18-induced stress. It was investigated whether the differential biosynthesis of flavonoids in the different *Arabidopsis* mutant lines could affect strain LB400 colonization ability and root adhesion pattern on a longer time frame. The Day 0 graph (**A**) shows the colonization efficiency of strain LB400 in plantlets germinated for 5 days in presence/absence of the bacterium in the agar medium (day 0 of the assay). It represents the baseline colonization of the plantlets before transferring them to new plates to test resistance to PCB-18 stress or to control plates with acetone. Graphs (**B**) and (**C**) represent LB400 root colonization efficiency on *Arabidopsis* plantlets respectively 7 or 14 days after the transfer on plates supplemented with acetone or 20 µM PCB-18. Strain LB400 showed similar levels of colonization in the different genotypes of *Arabidopsis*. The barplots represent data from three independent experiments. Statistical analysis was performed using one-way ANOVA.

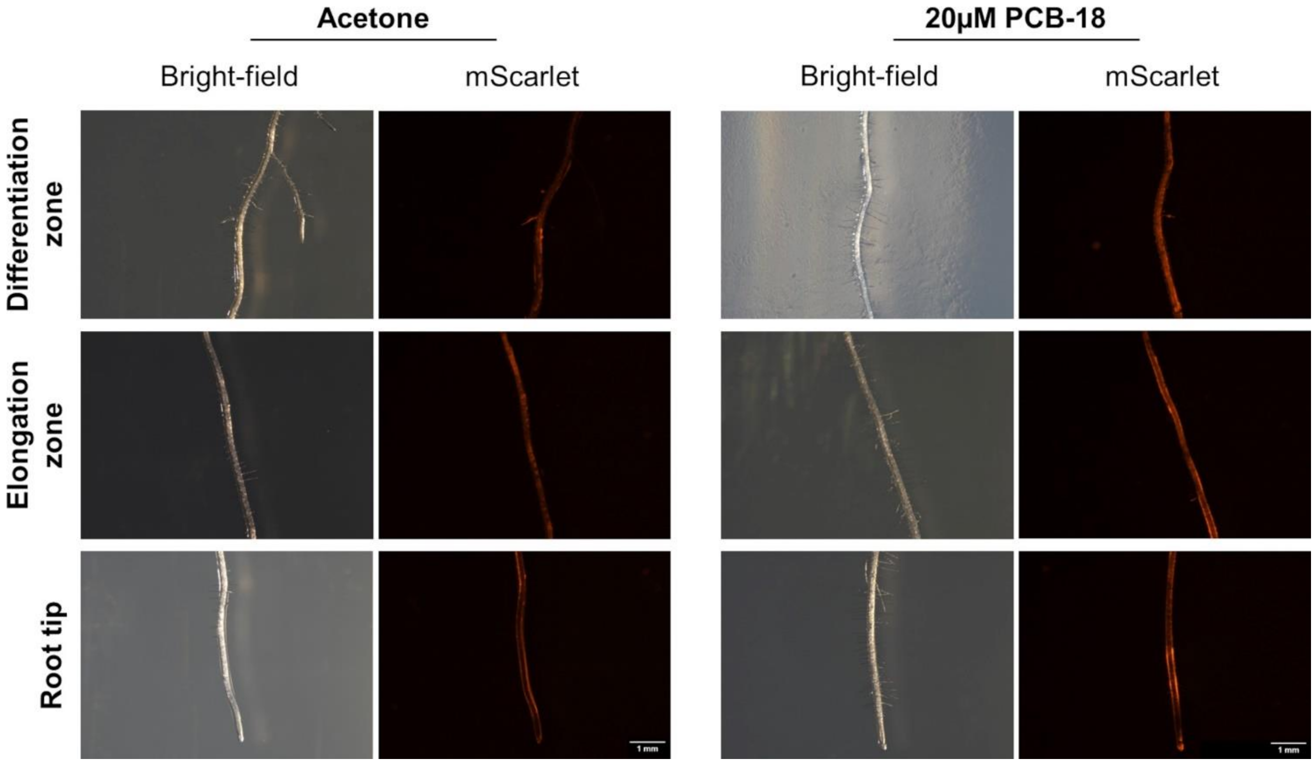

Supplementary Figure 9. Fluorescence microscopy analysis of *mScarlet*-tagged LB400 strain colonization profile on the root system of *Arabidopsis* WT. A fluorescent *mScarlet*-labelled *P. xenovorans* LB400 strain was developed in this study and fluorescence microscopy analyses were performed to elucidate the bacterial adhesion pattern in root colonization of the different *Arabidopsis* lines under control conditions (acetone) and in presence of 20 µM PCB-18. The panels illustrate the pattern of colonization in the different regions of the root system (differentiation zone, elongation zone and root tip). Plantlets were analyzed at 7 DAT and it was observed that *mScarlet*-labelled LB400 strain largely decorated *Arabidopsis* primary root and the root tip, while secondary roots were not colonized. These findings suggest that 7 days after transfer and exposure to PCB stress, *P. xenovorans* LB400 colonization patterns are not diversely affected in *Arabidopsis* mutant plantlets with distinct flavonoid exudation.

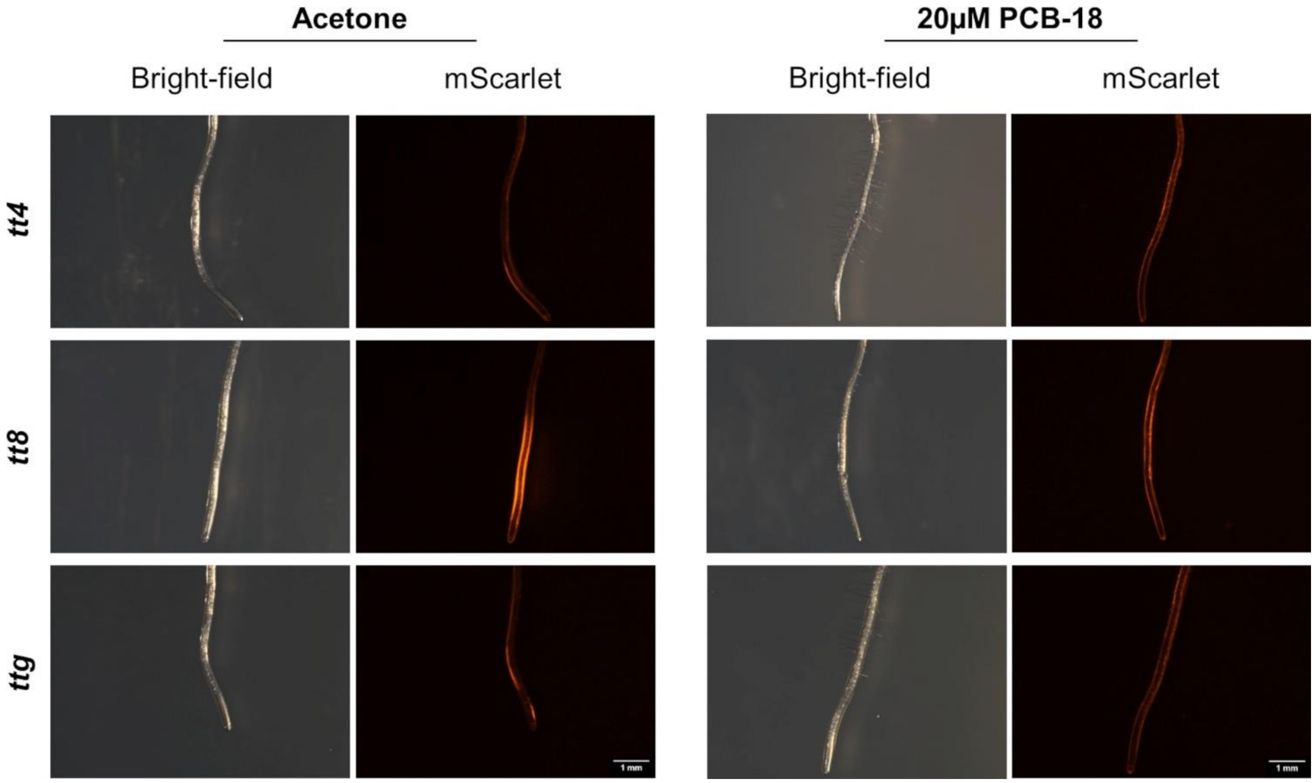

Supplementary Figure 10. Fluorescence microscopy analysis of *mScarlet*-tagged LB400 strain colonization profile on the apical region (root tip) of roots of flavonoids-affected *Arabidopsis* mutant lines *tt4*, *tt8* and *ttg* under control conditions (acetone) and under PCB-18 stress. The fluorescence microscopy analysis was performed to unravel *mScarlet*-tagged LB400 strain colonization pattern on the root system of the *Arabidopsis* lines affected in flavonoid biosynthesis and accumulation under control conditions (acetone) and in presence of 20 µM PCB-18. The panels illustrate the pattern of colonization in the root tip region for the *Arabidopsis* mutant lines *tt4, tt8* and *ttg*.

Treatment	Concentration (µM)	Maximum growth rate	Increase (%)	Entrance in stationary phase (hours)	OD_{600} at entrance in stationary phase
**Flavone**	20	$0.0206 \pm 0.0005^{***}$	+9.6%	18	0.1876
	50	0.0197 ± 0.0007^{ns}	+4.8%	18	0.1714
**Flavanone**	20	0.0214 ± 0.0008^{ns}	+2.4%	17	0.2034
	100	0.0205 ± 0.0012^{ns}	-1.9%	17	0.1913
**Naringin**	10	$0.0229 \pm 0.0006^{***}$	+6.0%	17	0.1781
	20	$0.0235 \pm 0.0001^{***}$	+8.8%	17	0.1830
	50	$0.0245 \pm 0.0009^{***}$	+13.4%	17	0.2077
	100	$0.0244 \pm 0.0009^{***}$	+13.0%	17	0.1989
**Naringenin**	50	$0.0272 \pm 0.0011^{***}$	+9.2%	17	0.2266
	100	0.0263 ± 0.0011^{ns}	+5.6%	17	0.2135
**Quercetin**	10	$0.0158 \pm 0.0022^{***}$	-20.2%	21	0.1891
	20	$0.0169 \pm 0.0001^{***}$	-14.6%	22	0.2100
	50	$0.0163 \pm 0.0004^{***}$	-17.7%	23	0.2238
	70	$0.0114 \pm 0.0005^{***}$	-42.4%	28	0.2048

Supplementary Table 1. Values of strain LB400 maximum growth rate when exposed to growth-stimulating plant flavonoids. In the table, the maximum growth rate was calculated for the flavonoid molecules and for the concentrations that induced a growth promotion as indicated in Figure 1 of the manuscript. The % increase in growth rate compared to the solvent control, the time of entrance in stationary phase and the OD_{600} reached in the moment of entrance in stationary phase are also reported. Statistical analysis was performed using the Mann-Whitney test, by comparing the flavonoid treatment with the respective solvent control. ***: $p \leq 0.001$; ns: non-significant ($p > 0.05$).
